# Plant phylogeny drives arboreal caterpillar assemblages across the Holarctic

**DOI:** 10.1002/ece3.7005

**Published:** 2020-11-07

**Authors:** Carlo L. Seifert, Martin Volf, Leonardo R. Jorge, Tomokazu Abe, Grace Carscallen, Pavel Drozd, Rajesh Kumar, Greg P.A. Lamarre, Martin Libra, Maria E. Losada, Scott E. Miller, Masashi Murakami, Geoffrey Nichols, Petr Pyszko, Martin Šigut, David L. Wagner, Vojtěch Novotný

**Affiliations:** ^1^ Institute of Entomology Biology Centre of the Czech Academy of Sciences České Budějovice Czech Republic; ^2^ Faculty of Science University of South Bohemia České Budějovice Czech Republic; ^3^ Faculty of Science Chiba University Chiba Japan; ^4^ Conservation Ecology Center Smithsonian Conservation Biology Institute Front Royal VA USA; ^5^ Faculty of Science University of Ostrava Ostrava Czech Republic; ^6^ Central Sericultural Research and Training Institute Central Silk Board Ministry of Textiles Govt. of India Pampore Jammu and Kashmir India; ^7^ ForestGEO Smithsonian Tropical Research Institute Balboa, Ancon Panama; ^8^ National Museum of Natural History Smithsonian Institution Washington DC USA; ^9^ University of Connecticut Storrs CT USA

**Keywords:** deciduous forests, feeding guilds, insect herbivores, Lepidoptera, phylogenetic isolation, shelter builders, specialization, species richness

## Abstract

1. Assemblages of insect herbivores are structured by plant traits such as nutrient content, secondary metabolites, physical traits, and phenology. Many of these traits are phylogenetically conserved, implying a decrease in trait similarity with increasing phylogenetic distance of the host plant taxa. Thus, a metric of phylogenetic distances and relationships can be considered a proxy for phylogenetically conserved plant traits and used to predict variation in herbivorous insect assemblages among co‐occurring plant species.
2. Using a Holarctic dataset of exposed‐feeding and shelter‐building caterpillars, we aimed at showing how phylogenetic relationships among host plants explain compositional changes and characteristics of herbivore assemblages.
3. Our plant–caterpillar network data derived from plot‐based samplings at three different continents included >28,000 individual caterpillar–plant interactions. We tested whether increasing phylogenetic distance of the host plants leads to a decrease in caterpillar assemblage overlap. We further investigated to what degree phylogenetic isolation of a host tree species within the local community explains abundance, density, richness, and mean specialization of its associated caterpillar assemblage.
4. The overlap of caterpillar assemblages decreased with increasing phylogenetic distance among the host tree species. Phylogenetic isolation of a host plant within the local plant community was correlated with lower richness and mean specialization of the associated caterpillar assemblages. Phylogenetic isolation had no effect on caterpillar abundance or density. The effects of plant phylogeny were consistent across exposed‐feeding and shelter‐building caterpillars.
5. Our study reveals that distance metrics obtained from host plant phylogeny are useful predictors to explain compositional turnover among hosts and host‐specific variations in richness and mean specialization of associated insect herbivore assemblages in temperate broadleaf forests. As phylogenetic information of plant communities is becoming increasingly available, further large‐scale studies are needed to investigate to what degree plant phylogeny structures herbivore assemblages in other biomes and ecosystems.

## Graphical abstract

Host plant phylogenetic relationships have been shown to affect community composition and interaction networks of associated insect herbivores. Using a cross‐continental dataset, we showed that phylogenetically isolated hosts harbored less diverse and less specialized caterpillar assemblages. Our results suggest that a combination of phylogenetic information and compositional structure of the host plant community provides useful measures to predict the structure of herbivore assemblages among coexisting plant species.

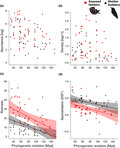

## 1 INTRODUCTION

Plants have evolved a variety of physical, chemical, and life history traits in order to avoid or lessen herbivory by insects. A range of plant traits, such as trichomes, leaf toughness, latex and resin, secondary metabolites, or volatile organic compounds, act directly or indirectly as defense mechanisms against insect herbivores (Agrawal, 2007; Agrawal & Hastings, 2019; Carmona et al., 2011; Clissold et al., 2009; Lämke & Unsicker, 2018). Further plant traits, such as nutrient content and life history traits (e.g., phenology), influence herbivore development, fecundity, and performance (Awmack & Leather, 2002; Segarra‐Carmona & Barbosa, 1983; Wetzel et al., 2016). In order to overcome such defense mechanisms and exploit resources of varying quality, insect herbivores have evolved myriad morphological, biochemical, life history, and behavioral adaptations (War et al., 2018). Since plant traits are, to a greater or lesser extent, phylogenetically conserved (Agrawal, 2007; Davies et al., 2013; Larose et al., 2019; Rønsted et al., 2012; Whitfeld et al., 2012), these counterdefense adaptations often result in resource specialization constrained by plant phylogeny (Jorge et al., 2014, 2017; Nipperess et al., 2012; Ødegaard et al., 2005; Wang et al., 2020; Weiblen et al., 2006). Moreover, metrics indicating phylogenetic relationships among plant species could be used as a “proxy measure” for trait similarity (Endara et al., 2017; Pearse & Hipp, 2009; Pellissier et al., 2013). As it is infeasible to measure all plant traits that potentially influence insect herbivores, phylogenetic metrics might thus provide a promising surrogate to explain and predict the nature of a host's herbivore fauna, for example, its richness, taxonomic composition, collective abundances, or degree of dietary specialization (Wang et al., 2020).

Lavandero et al. (2009), for instance, showed that herbivore specialization increases with community‐wide uniqueness of host plant chemistry. This uniqueness in defensive traits, such as secondary metabolites, in turn increases with phylogenetic distance to the closest related species (Larose et al., 2019; Rønsted et al., 2012), leading to the prediction of higher specialization of herbivore assemblages with increasing phylogenetic isolation of the host plant. A recent study by Grandez‐Rios et al. (2015) indeed reported such a positive correlation.

Moreover, increased trait overlap among closely related plant species facilitates host shifts for insect herbivores, which are adapted to the shared plant traits (Pearse & Altermatt, 2013; Pearse et al., 2013). Consequently, plant taxa with co‐occurring relatives (e.g., congeners) provide more potential food resources for consumers such as specialized herbivorous insects. A decrease in phylogenetic isolation is thus expected to increase the richness of the associated insect herbivore assemblage, as predicted by the *taxonomic isolation hypothesis* (see Brändle & Brandl, 2001; Connor et al., 1980; Kennedy & Southwood, 1984). This hypothesis has been supported by several large‐scale studies (Grandez‐Rios et al., 2015; Joy & Crespi, 2012; Lin et al., 2015), and it could also be expected to apply when local communities are the focus of investigation (Vialatte et al., 2010).

Insect herbivore abundance is often positively correlated with species richness (Bock et al., 2007; Zanuncio et al., 2001); however, compared with richness, the influence of phylogenetic isolation on abundance of associated insects is less clear and in need of further investigation. Vialatte et al. (2010) reported that increasing phylogenetic isolation led to lower abundances of tree‐dwelling insects. In contrast, an extensive study accounting for resource availability in tropical forests of Papua New Guinea by Whitfeld et al. (2012) revealed that the effect of plant phylogeny on herbivore abundance did not account for appreciable variation, whereas leaf biomass proved to be a strong predictor of herbivore abundance. As would be expected by the *resource availability hypothesis* (see Herms & Mattson, 1992; Wardhaugh, 2014), and confirmed by various studies (Scherrer et al., 2010; Seifert et al., 2019; Whitfeld et al., 2012), resource abundance is frequently found to be a strong predictor of insect herbivore abundance. Moreover, Root’s (1973) *resource concentration hypothesis* extends the *resource availability hypothesis* as it not only assumes an increase in herbivore abundance with increasing foliage area (concentration) of a given host, but also predicts an increase in herbivore density. Thus, derived from its assumptions, abundant plant species should harbor not only more herbivores than rare species, but also higher numbers of individuals per unit of leaf area. Accordingly, plant species that provide much of the foliage area would be expected to harbor higher loads of associated insects.

Comprising more than 157,000 described species worldwide (van Nieukerken et al., 2011), the insect order Lepidoptera is one of the largest single radiations of plant‐feeding insects on earth (Menken et al., 2010; Mitter et al., 2017). Their larval stages represent a broad range of resource specialization and comprise a variety of different feeding guilds such as stem borers, root feeders, miners, gallers, shelter builders, or exposed feeders (Gaston et al., 1992). These feeding guilds have profound ecological implications on diet breadth, composition, and diversity. For example, shelter‐building caterpillars are commonly smaller and more specialized than exposed feeders (Gaston et al., 1992; Menken et al., 2010). While exposed feeders often reveal a higher richness, shelter builders widely dominate local caterpillar communities (Diniz et al., 2012; Hrcek et al., 2013). Shelter builders actively manipulate plant parts of their hosts, which was shown to improve resource quality by lowering physical and chemical plant defenses (Sagers, 1992). Exposed feeders, by contrast, are capable to forage selectively due to their higher mobility.

The remarkable taxonomic richness of Lepidoptera, their ecological diversity, and species radiations across the phylogeny of vascular plants make them especially well‐suited to explore the ecological patterns and mechanisms likely to be common to insect–plant associations across the Tree of Life.

Large‐scale studies from different biogeographical regions allow the detection of global ecological patterns. Here, we explore to what degree the phylogenetic relatedness of host trees can be used to predict compositional turnover, as well as taxonomic richness, abundance, density, and degree of dietary specialization of a host's herbivore fauna. Our data are drawn from three temperate forest ecosystems of the Northern Hemisphere, spanning three continents: Asia, North America, and Europe.

We use an extensive intercontinental dataset of plant–herbivore interactions to investigate the influence of plant phylogenetic isolation and relatedness on herbivore assemblages by testing the following four hypotheses:

1. Overlap (similarity) of caterpillar assemblages decreases with increasing phylogenetic distance of the host plant species.
2. Caterpillar abundance and density are driven by foliage availability rather than by plant phylogeny in accordance with the *resource availability hypothesis* and the *resource concentration hypothesis*, respectively.
3. Caterpillar richness decreases with increasing phylogenetic isolation as predicted by the *taxonomic isolation hypothesis*.
4. Mean dietary specialization of caterpillar assemblages increases with increasing phylogenetic isolation.

## 2 MATERIALS AND METHODS

### 2.1 Sampling

Sampling was conducted in three temperate lowland forests: Tomakomai (Hokkaido, Japan; 42°43′N, 141°36′E; 90 m a.s.l.), Toms Brook (Virginia, USA; 38°55′N, 78°25′W; 220 m a.s.l.), and Lanžhot (Czech Republic; 48°42′N, 16°57′E; 152 m a.s.l.). All sites comprise lowland broadleaf forest communities typical for the given regions. Non‐native vegetation and disturbed areas were avoided. Only woody plants with DBH ≥ 5 cm were sampled; tree ID, tree abundance, leaf area, and number of sampled caterpillars are given in Table 1. The Czech site had the lowest plant richness (8 host plant species), followed by the USA (15 species) and Japan (20 species). The Czech and Japanese sites were dominated by *Acer* L. (Sapindaceae), *Fraxinus* L. (Oleaceae), and *Carpinus* L. (Betulaceae). In the USA, *Quercus* L. (Fagaceae), *Nyssa* gronov. ex. l. (Cornaceae), and *Carya* nutt. (Juglandaceae) were the most abundant genera (Table S1).

**Table 1:** Characteristics of the three sampling sites (two 0.1‐ha plots each) including climate, plant, and caterpillar abundance and diversity, as well as sampled leaf area

	Czech Republic	Japan	USA
Climate			
Mean annual temperature (°C)	9.0	5.6	12.9
Mean annual precipitation (mm)	525	1,161	1,000
Plants			
*N*_Ind_|*N*_Spp_|*N*_Fam_	56|8|6	185|20|12	161|15|9
Total leaf area (m^2^)	2,417	2,378	3,586
Caterpillars			
Total: *N*_Ind_|*N*_Spp_|*N*_Fam_	8,573|107|20	15,511|148|21	3,954|141|20
Exp.: *N*_Ind_|*N*_Spp_|*N*_Fam_	7,625|69|10	10,275|83|10	1,575|82|11
She.: *N*_Ind_|*N*_Spp_|*N*_Fam_	948|38|10	5,236|65|12	2,379|59|10

Note

The number of individuals (*N*_Ind_), species (*N*_Spp_), and families (*N*_Fam_) included in the analyses are given for the plant and the caterpillar communities. The caterpillar data are further separated into feeding guilds (Exp. = exposed feeders, She. = shelter builders).

We employed plot‐based sampling, following the approach and protocols described in Volf et al. (2019), to investigate insect–plant interactions and the assemblage structure of insect herbivores. At each site, we set up two 0.1‐ha plots and sampled caterpillars from all deciduous trees with a DBH ≥5 cm. The sampling was carried out during the vegetation seasons between 2013 and 2017, but usually finished within two years at a given site (Czech Republic: May 2013–April 2015; Japan: May 2014–July 2015; USA: April 2016–August, 2017).

The trees were sampled either by felling (USA), by using a canopy crane (Japan), or by using a mobile elevating work platform (Czech Republic). To capture seasonal changes in species composition, the sampling of conspecific tree individuals was spread across the vegetation period.

Caterpillars were sampled manually from accessible foliage, assigned a morphotype and unique number, reared, and subsequently identified. Each caterpillar species was assigned to a guild as either being an exposed feeder (living free on the foliage = physically unprotected) or shelter builder (i.e., leaf roller, leaf tier, and webber). Species identifications were based on external morphological characteristics of caterpillars and reared adults, and verified by comprehensive DNA barcoding (i.e., sequencing of a 658‐bp fragment of the COI gene; Ratnasingham & Hebert, 2013). Adult identification was further aided in many cases by genitalia dissections. Detailed protocols for morphotyping, rearing, and identification can be found in Volf et al. (2019). Voucher specimens from Czech Republic and Japan are deposited at the University of Ostrava and the University of Chiba, respectively. Specimens from the USA are stored at the Institute of Entomology in Ceske Budejovice (larvae) and the Smithsonian National Museum of Natural History, Washington, D.C. (adults).

To quantify resource availability, we estimated the leaf area of each tree we sampled. In order to achieve this, we defoliated each tree individual (small trees: 100%; mid‐size trees: 50%; large trees: 25%) and calculated its total leaf biomass. We then took a random subsample of these leaves, photographed, and weighted them to calculate the weight‐to‐leaf area ratio for this subsample. Afterward, we calculated the total area of sampled leaves (m^2^) for each tree by extrapolating the weight‐to‐leaf area ratio of the photographed subsample by the total leaf biomass (for detailed protocols, see Volf et al., 2019). In order to indicate the availability (i.e., leaf area) of a given host plant within its community, individual‐based estimations were summed for each tree species.

### 2.2 Plant phylogenetic isolation and relatedness

**Figure 1:**
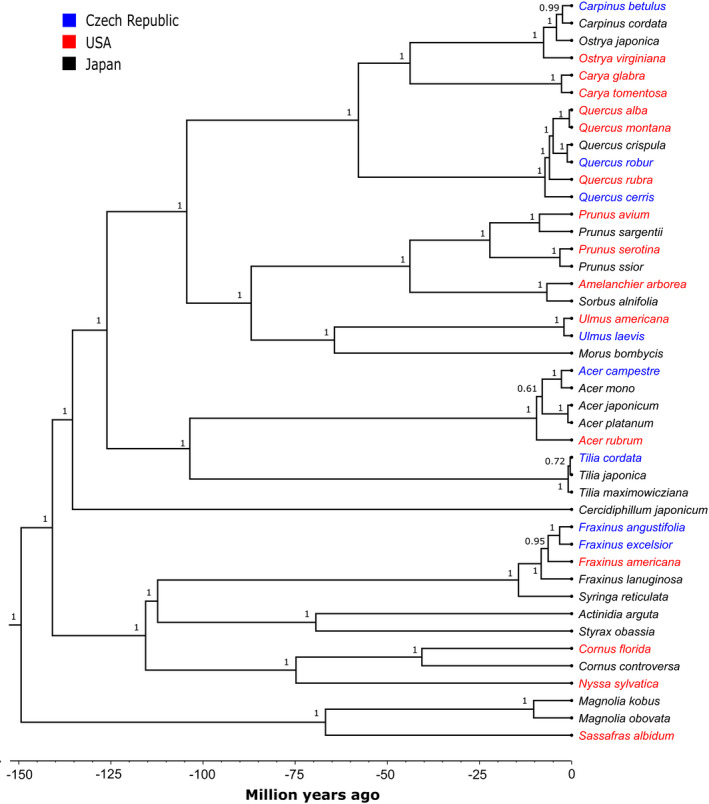
Host plant phylogeny including all tree species that were sampled at our three study sites (Czech Republic, USA, and Japan). Posterior probabilities are given for each node. The topology was constrained, and a log‐normal relaxed molecular clock was used for time calibration

We reconstructed a phylogeny for all focal plant species using four loci: *rbcL*, *matK*, *ITS*, and *trnL‐trnF*. Sequences for the studied tree species were either extracted from Volf et al. (2017; accessible at: www.ebi.ac.uk/ena/data/view/LT671631‐LT671669) or downloaded from GenBank (Table S2). The sequences were edited and aligned in Geneious 5.4 (Drummond et al., 2011). A host plant phylogeny was reconstructed using the Bayesian inference as implemented in BEAST v2.4 (Drummond et al., 2012). The following substitution models for individual loci were selected based on BIC computed in jModelTest 2 (Darriba et al., 2012): *rbcL*: TPM3uf + I + G, *matK*: TVM + I+G, *ITS*: GTR + G, and *trnL‐trnF*: TPM1uf + G. The topology was constrained using Phylomatic 3 (Webb & Donoghue, 2005). We used additional constraints for *Quercus* based on Denk et al. (2017). We did not constrain inner topology of *Ostrya* and *Carpinus* as monophyly of these genera remains uncertain (see Grimm & Renner, 2013; Yang et al., 2019). A log‐normal relaxed molecular clock with dating following Bell et al. (2010) was used for time calibration (millions of years). Sampling was carried out every 10^3^ generations for 3x10^7^ generations, the first 10% of all generations were discarded as “burn‐in,” and the results were summarized with a majority‐rule consensus tree. The time‐calibrated master phylogeny (Mya; Figure 1) was pruned into three “subphylogenies,” each of them comprising the respective species pool of a given site (i.e., Czech Republic, Japan, USA).

*Quercus rubra* L. and *Quercus velutina* Lam. often hybridize at the studied site in the USA. Thus, we treated them as a species complex hereafter referred to as *Quercus rubra* agg. We used the phylogenetic position of *Quercus rubra* for the *Quercus rubra* agg complex in all further analyses.

Matrices of phylogenetic distances among each pair of plant species were calculated as the patristic distances using the *distTip* function implemented in the “*adephylo”* package (Jombart et al., 2010). To measure the phylogenetic isolation of a given tree species within its community, we calculated the “evolutionary distinctiveness” applying fair proportions (Isaac et al., 2007) by using the package “*picante”* (Kembel et al., 2010). Evolutionary distinctiveness indicates a species’ isolation within a given phylogenetic tree. High scores of evolutionary distinctiveness indicate higher phylogenetic isolation, whereas low scores indicate the presence of proximate relatives. Both plant phylogenetic distances and plant phylogenetic distinctiveness were calculated separately for each sampling site. Hereafter, evolutionary distinctiveness is referred to as “*Phylogenetic isolation”* (PI).

### 2.3 Statistical analyses

Before we tested our hypotheses, all caterpillar species sampled in less than three individuals were excluded from the dataset in order to eliminate erroneous interaction records and to avoid overestimating caterpillar specialization. Two resource‐level measures, phylogenetic isolation and availability (i.e., leaf area) of the host plant species, were used as predictors to test our hypotheses on caterpillar abundance, density, richness, and specialization. In order to assess the independence between these predictors and drive the interpretation of our results, we tested for a relationship between these variables using a least squares linear model. Leaf area was used as dependent variable and square‐root‐transformed to meet the assumption of normality. Phylogenetic isolation was used as an independent variable. To account for intersite differences, sampling site was included as a covariate.

#### 2.3.1 The influence of plant phylogenetic distance on similarity of caterpillar assemblages

In order to test our hypothesis that caterpillar assemblage similarity decreases with increasing phylogenetic distance of the host plants, we used the modified Mantel tests. Due to a lack of shared plant and caterpillar species among sites, the creation of a global matrix (i.e., pairwise distances between the trees of all sites) was not appropriate. Nor did it make sense to test for differences between study sites. Therefore, in order to avoid any regional bias, and to avoid including spurious distance values between the host plant species of different sites, we implemented the following modifying protocol:

- Plant phylogenetic distance (patristic distances between pairs of host plants) and caterpillar species overlap matrices (using Jaccard's similarity) were created separately for each site.
- We then combined or concatenated the three matrix pairs together to obtain the single observed correlation coefficient.
- Similarly, null correlations were obtained by first randomizing the phylogenetic distance matrices for each site, then combining them prior to calculating the correlation.
- The null model was run for 9,999 iterations and compared with the observed value.

We believe the modifications are justified in this situation, given that we are working with simple Mantel tests with no partial effects, and the two matrices are intrinsically distances and similarities, instead of raw variables (Legendre & Fortin, 2010).

The modified Mantel tests were performed across guilds and separately for exposed feeders and shelter builders. Additional to the Jaccard's similarity, we also conducted the modified Mantel tests based on caterpillar abundance data by using Bray–Curtis and Morisita–Horn similarity indices.

#### 2.3.2 The effect of plant phylogenetic isolation on caterpillar abundance and density

First, we tested the *resource availability hypothesis*, which predicts that caterpillar abundance is primarily driven by resource availability (i.e., leaf area) rather than by host phylogenetic isolation. Caterpillar abundance was measured as number of caterpillar individuals (log‐transformed) associated with a particular tree species. We then fitted a set of linear mixed models (LMMs) with “*Abundance”* as response variable and “*Phylogenetic isolation*” and “*Feeding guild”* as fixed effects. Additionally, “*Leaf area”* (square‐root‐transformed) and “*Sampling site”* were considered fixed effects to account for variations in resource availability among host plants and regional differences. “*Tree identity”* was used as a random effect to account for the nonindependence of the results from the different feeding guilds in the same host tree.

Second, we tested the *resource concentration hypothesis*, which predicts that caterpillar densities increase with available foliage area of the host tree species. Caterpillar density values were calculated as the total number of caterpillars · m^‐2^ leaf area for each tree species and (log + 1)‐transformed prior to analyses. Afterward, we fitted a set of LMMs by using the same predictors as for the models on abundance.

For both response variables “*Abundance”* and “*Density*,” we conducted models representing all possible combinations of factors additively, also including the interactions between “*Feeding guild”* and “*Sampling site.”* Afterward, we used the corrected Akaike information criterion (AICc) and their weights (*w*) to rank and select the best models (Burnham & Anderson, 2002). Additionally, we tested for the significance of the best‐fitting models (ΔAICc ≤ 2 and least number of parameters) by means of chi‐square likelihood‐ratio tests between each of them and the respective null model including only the random effect and a fixed intercept. Tukey post hoc tests were further applied on the best model for each response variable to test for differences among feeding guilds at each sampling site.

#### 2.3.3 The effect of plant phylogenetic isolation on caterpillar richness

Here, we tested the *taxonomic isolation hypothesis*, which predicts that caterpillar richness declines with increasing phylogenetic isolation of the host plant. Caterpillar richness was measured as the number of caterpillar species associated with a given plant species. We then conducted a set of LMMs with “*Richness”* as response variable, and “*Phylogenetic isolation*,” “*Feeding guild*,” “*Leaf area”* (square‐root‐transformed), and “*Sampling site”* as fixed effects. “*Tree identity”* was used as a random effect to account for the nonindependence of exposed feeder and shelter builder richness in the same host tree. The models represented all possible combinations of factors additively, also including the interaction between “*Feeding guild”* and “*Sampling site.”* We then used the corrected Akaike information criterion (AICc) and their weights (*w*) to select the best model (Burnham & Anderson, 2002). The best model (ΔAICc ≤ 2 and least number of parameters) was further compared with the null model including only the random effect and a fixed intercept by means of chi‐square likelihood‐ratio test. A Tukey post hoc test was further applied on the best model to test for differences among feeding guilds at each sampling site.

#### 2.3.4 The effect of plant phylogenetic isolation on specialization

Here, we tested our hypothesis that mean specialization of caterpillar assemblages increases with increasing phylogenetic isolation of the host plant species. Specialization was measured at the herbivore species level using the standardized distance‐based specialization index (DSI*; Jorge et al., 2014, 2017). This index takes resource phylogenetic relatedness and availability, and herbivore frequency into account. It thus reflects the specialization of consumer species more mechanistically than traditional indices and further allows for robust comparisons (Jorge et al., 2014, 2017).

The DSI*_i_* (Jorge et al., 2014) in its nonstandardized form is calculated as follows:

$${\text{DSI}}_{i}=\left(\frac{{\text{MPD}}_{i}‐\text{mean}{\left(\text{Null}\right)}_{i}}{\text{sd}{\left(\text{Null}\right)}_{i}}\right)\left(‐1\right),$$

where MPD*_i_* is the mean pairwise phylogenetic distance of hosts utilized by caterpillar species *i*, weighted by their host abundances within the plant community (i.e., m^2^ leaf area). Null*_i_* is the random mean pairwise phylogenetic distance calculated separately for each herbivore species. This is done by randomly selecting a set of hosts with the same size as the interaction frequency of a given herbivore. This set is sampled from the host plant pool, which is available to caterpillar species *i*, taking resource abundance (i.e., leaf area) and occurrence into account. Thus, total leaf area (m^2^) of the tree species within the respective plant community was used as indicator for its availability. The tree phylogenies, pruned for each site, were used to account for resource relatedness.

The standardized distance‐based specialization index (DSI*, Jorge et al., 2017) represents a rescaled version of the DSI, and its value ranges between −1 (maximum generalization) and 1 (maximum specialization). DSI* is calculated as:

$${\text{DSI}}_{i}^{*}=\frac{{\text{DSI}}_{i}}{\left|{\text{DSI}}_{\lim}\right|},$$

where DSI_lim_ is the minimum (for negative DSI*_i_*)/ or maximum (for positive DSI*_i_*) possible DSI value for a given caterpillar species *i*.

DSI* values were calculated separately for each sampling site and assigned to each caterpillar species. Afterward, for every host plant species the mean DSI* values of its associated shelter‐building and exposed‐feeding caterpillar assemblages were calculated. By accounting for resource availability and herbivore frequency, and relatedness, DSI* is not numerically dependent on phylogenetic isolation. Additionally, we calculated an average DSI* for the caterpillar fauna feeding on each plant species, which further allows for variation in DSI* independently from phylogenetic isolation of the host plant.

In order to test whether mean caterpillar specialization increases with phylogenetic isolation of the host plant, we conducted a set of models with “*Phylogenetic isolation*,” “*Feeding guild*,” “*Leaf area”* (square‐root‐transformed), and “*Sampling site”* as fixed effects. This set of models represented all possible combinations of factors additively, including the interaction between “*Feeding guild”* and “*Sampling site.” “Tree identity”* was used as a random effect to account for the nonindependence in specialization of exposed feeders and shelter builders within the same host tree. We then used the corrected Akaike information criterion (AICc) and their weights (*w*) to select the best model (ΔAICc ≤ 2 and least number of parameters; Burnham & Anderson, 2002), which was further compared with the respective null model including only the random effect and a fixed intercept using a likelihood‐ratio test on chi‐square distribution. A Tukey post hoc test was applied on the best model for DSI* to test for differences among feeding guilds at each sampling site.

All statistical analyses were performed using the software R v. 4.0.2 (R Development Core Team, 2020). Calculations of similarity indices were conducted using the “*vegan”* package (Oksanen et al., 2018). LMMs were generated using the “*lme4”* package (Bates et al., 2015).

## 3 RESULTS

Our final dataset comprised more than 28,000 discrete caterpillar–plant interactions and 382 lepidopteran species from 32 families (Table 1, Table S3). Each sampling site had a unique set of host plants, and only 13 caterpillar species were found in more than one locality (Table S3). Thus, our study sites differed essentially at the level of interacting species pools. We found a negative relationship between resource availability and phylogenetic isolation of the studied plant taxa (*R*_adj_ *^2^* = 0.22, *F*_3,39_ = 4.92, *p* = .005, *n* = 43), indicating that phylogenetically isolated tree species provide less foliage area.

### 3.1 The effect of plant phylogenetic distance on caterpillar species overlap

**Figure 2:**
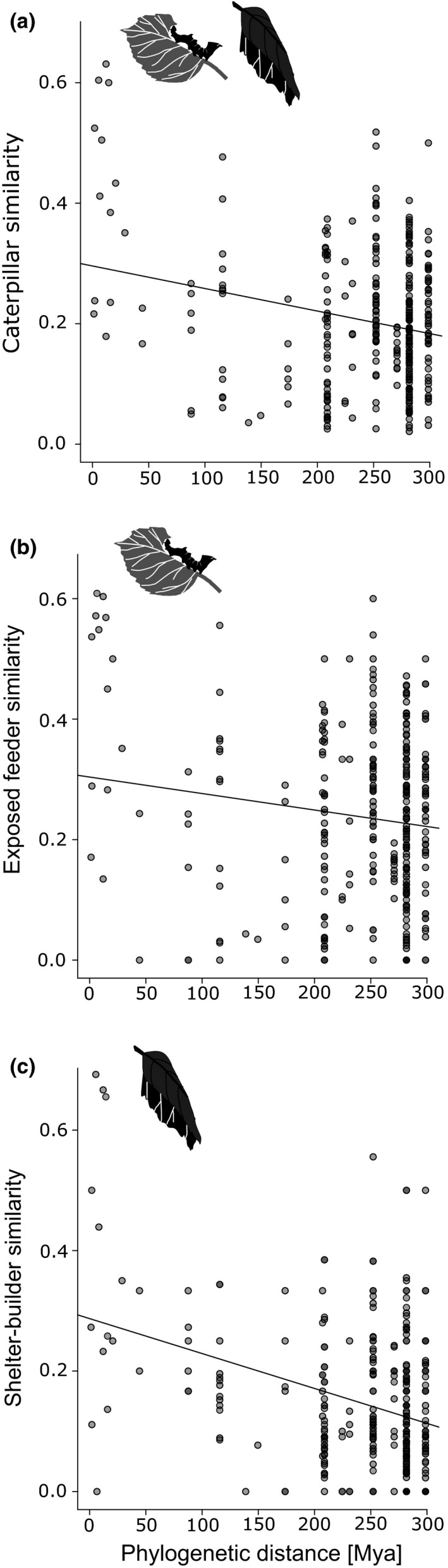
The influence of phylogenetic distance on caterpillar species overlap based on Jaccard's similarity (a) across feeding guilds (*r* = −0.23, *p* < .001), as well as for (b) exposed feeders (*r* = −0.13, *p* < .001) and c) shelter builders (*r* = −0.34, *p* < .001). P‐values were obtained from Mantel tests modified to account for sampling site (9,999 permutations)

Jaccard's similarities of caterpillar assemblages among each pair of host plant species were found to be generally low (mean ± *SD*: 20.5% ± 11.3%), indicating that the tree communities harbored largely unique assemblages of associated caterpillars. There was a negative relationship between phylogenetic distance of host plants and similarity of the associated caterpillar assemblages (*r* = −0.23, *p* < .001; Figure 2a), which was retained when exposed feeders and shelter builders were analyzed separately. Exposed‐feeding caterpillars, however, were less strongly affected by phylogenetic distance (*r* = −0.13, *p* < .001; Figure 2b) than shelter builders (*r* = −0.34, *p* < .001; Figure 2c). Analyses using the abundance‐based Bray–Curtis and Morisita–Horn indices revealed results similar to those found with the incidence‐based Jaccard index (Table S4).

### 3.2 The effect of plant phylogenetic isolation on caterpillar abundances and density

**Figure 3:**
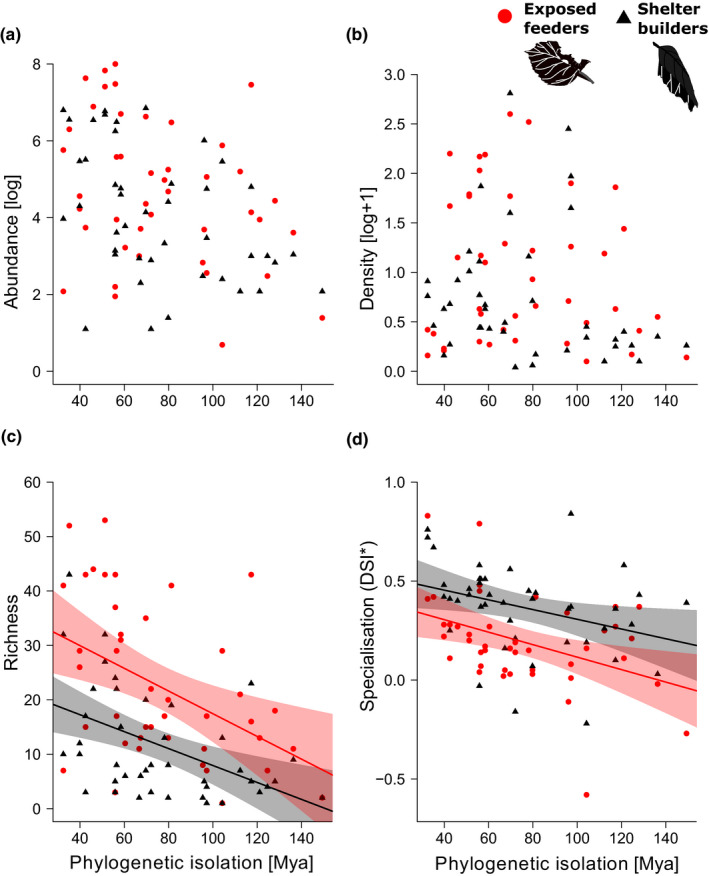
Guild‐specific relationships between plant phylogenetic isolation and (a) abundance, (b) density, (c) species richness, and (d) mean specialization of associated caterpillar assemblages. When LMMs indicated significance, regression lines were derived from simple linear regressions including only phylogenetic isolation as predictor variable (see Table 2)

Caterpillar assemblages of exposed feeders were significantly more abundant and reached higher densities per tree species compared with assemblages of shelter‐building caterpillars, although at the eastern USA site, no significant differences were observed among the guilds (Figure S1a, b). Caterpillar abundance was positively influenced by foliage area (resource availability) but not by phylogenetic isolation of the host (Table 2). For caterpillar density, neither phylogenetic isolation nor leaf area were included in the best model, indicating that herbivore densities were not influenced by phylogenetic position of the host plant species nor the host's commonness (measured as leaf area). These findings were consistent for both feeding guilds (Figure 3a, b; Table 2).

**Table 2:** Selected linear mixed models (LMMs) on abundance, density, richness, and mean specialization (DSI*) of caterpillar assemblages

Fixed effects	Abundance	Density	Richness	Specialization
	*df =9,* χ^2^ = 89.26***	*df =8,* χ^2^ = 48.87***	*df=10,* χ^2^ = 140.4***	*df=9,* χ^2^ = 45.31***
Intercept	0.944 ± 0.454	0.266 ± 0.186	−1.297 ± 4.125	0.528 ± 0.103
Phyl. isolation	–	–	−0.064 ± 0.028	−0.003 ± 0.001
Leaf area	0.173 ± 0.018	–	1.293 ± 0.115	–
Site				
Japan	1.862 ± 0.435	0.801 ± 0.220	6.015 ± 2.625	0.079 ± 0.084
USA	0.779 ± 0.442	0.217 ± 0.230	−0.099 ± 2.610	0.071 ± 0.087
Guild				
Exposed	2.269 ± 0.375	0.910 ± 0.218	15.625 ± 2.118	0.045 ± 0.068
Site:Guild				
Japan:Exposed	−1.470 ± 0.444	−0.487 ± 0.258	−3.625 ± 2.506	−0.329 ± 0.080
USA:Exposed	−2.751 ± 0.464	−1.086 ± 0.269	−9.025 ± 2.623	−0.190 ± 0.084

Note

For each response variable, the best model is shown along with coefficient estimates (±SE) of its predictor variables. Tree identity was included as a random effect. For each model, chi‐square and *p*‐values were obtained from an ANOVA where the model of interest was compared against the respective null model including only the random effect. In all cases where other models were within 2 units of AICc, they were more complex than the selected model (see Table S5 for the performance of all candidate models). Significance codes: *<.05; **<.01; ***<.001.

### 3.3 The effect of plant phylogenetic isolation on caterpillar richness

Assemblages of exposed caterpillars were significantly more species‐rich than those of shelter‐building caterpillars (Fig. S1c), a pattern that held across sampling sites. Increasing phylogenetic isolation of the host plant species led to a significant decline in species richness of the associated caterpillar assemblage. This negative effect was observed for both feeding guilds (Figure 3c, Table 2). Additionally, leaf area was included in the best‐fitting model, indicating that resource availability positively affected species richness (Table 2).

### 3.4 The influence of phylogenetic isolation on caterpillar dietary specialization

Mean specialization of caterpillar assemblages varied among the study sites (Fig. S1d, Table 2), and assemblages of exposed feeders were found to be generally less specialized than those of shelter builders (mean ± *SD*: DSI*_Exposed_ = 0.192 ± 0.24; DSI*_Shelter_ = 0.366 ± 0.22), except for the Czech Republic, where there was no significant difference in mean specialization between exposed feeders and shelter builders (Figure S1d, Table 2). The mean specialization of caterpillar assemblages significantly declined with increasing phylogenetic isolation of their host plant taxa (Table 2). This negative relationship was present for both exposed feeders and shelter builders (Figure 3d, Table 2).

## 4 DISCUSSION

By using a comprehensive, cross‐continental (Holarctic) dataset of more than 28,000 caterpillar–host plant interactions, our study clearly revealed that phylogenetic isolation of host plants negatively affects richness and mean specialization of associated insect herbivores but not their abundance or density. Furthermore, the species composition of caterpillar assemblages becomes increasingly dissimilar with increasing phylogenetic distance. These general patterns were found to be equally valid for exposed and shelter‐building caterpillar assemblages. We extended previous studies (Grandez‐Rios et al., 2015; Vialatte et al., 2010) by including resource availability (leaf area) in our models and by using a specialization index based on resource (host plant) relatedness, which in sum provides new insights into how plant phylogeny structures insect herbivore assemblages.

### 4.1 Does phylogenetic distance predict species turnover?

As hypothesized, increased phylogenetic distances among host plant species resulted in a higher dissimilarity among both exposed‐feeding and shelter‐building caterpillar assemblages. Stated differently, phylogenetically related plant species are more likely to harbor a similar caterpillar fauna than are more distantly related hosts. The use of closely related hosts results from phylogenetic conservatism in plant traits (Weiblen et al., 2006; Whitfeld et al., 2012; Winkler & Mitter, 2008) and has long been recognized to be widespread in herbivorous insects (Brändle & Brandl, 2006; Cirtwill et al., 2020; Dinnage et al., 2012; Jorge et al., 2014, 2017; Morais et al., 2011; Nipperess et al., 2012; Ødegaard et al., 2005; Wang et al., 2020; Weiblen et al., 2006, but see Endara et al., 2017). Many classes of plant secondary metabolites known to have roles in discouraging herbivory are restricted to a monophyletic group of plants (Agrawal, 2007). Likewise, many specialized insect herbivores are adapted to specific secondary metabolites and thus develop only on related plants producing these compounds (Braby & Trueman, 2006; Fordyce, 2010; Hernández‐Vera et al., 2019; Nylin et al., 2014).

Especially in temperate and desert regions, another important driver of host conservatism in herbivores could be the influence of phylogenetic conservatism in plant phenology (Davies et al., 2013). For instance, many spring‐active folivorous caterpillars are adapted to feed on new foliage and will fail if none or merely older leaves are available (Forkner et al., 2008; Pearse & Karban, 2013). Phenological mismatches, for example, hatching from eggs or breaking diapause for those taxa overwintering as larvae, either too early or too late, would directly result in reduced caterpillar fitness and increased mortality (see van Asch & Visser, 2007, and references therein). To what degree phenological asynchrony affects species differently depending on their degree of dietary specialization needs further investigation.

### 4.2 Does phylogenetic isolation affect caterpillar abundances and density?

As hypothesized, our results supported the *resource availability hypothesis* (Herms & Mattson, 1992; Wardhaugh, 2014), as the interspecific variation in overall and guild‐specific caterpillar abundances was mainly explained by the leaf area of a given tree species. Moreover, phylogenetic isolation was not included in the best‐fitting model, which indicates that caterpillar abundances were randomly distributed across the host plant phylogeny at the three forest sites. This is in line with results from caterpillar assemblages in primary and secondary forests of Papua New Guinea (Whitfeld et al., 2012), where leaf biomass was the best predictor for caterpillar abundances, while other functional plant traits played a minor role. Vialatte et al. (2010), by contrast, found lower abundances of Heteroptera with increasing phylogenetic isolation of oaks in an European temperate forest. This contrasting finding most likely resulted from the fact that Heteroptera comprises various dietary guilds (predators, omnivores, and phytophages), and thus, comparisons with strict herbivores such as folivorous caterpillars might be expected to be inconsistent.

Based on assumptions of the *resource concentration hypothesis*, we predicted not only caterpillar abundance, but also densities to increase with foliage availability for a given host tree, whereas no effect of phylogenetic isolation was expected. Phylogenetic isolation, indeed, was found to have no significant effect on caterpillar density, nor did we find an effect of resource availability. This, on the one hand, underpins the strong correlation between caterpillar abundance and foliage availability (i.e., leaf area). On the other hand, it indicates that caterpillar densities were not influenced by commonness or rarity of a tree species.

From a plant perspective, caterpillars represent the dominant guild of leaf chewing insects in temperate forest ecosystems. Although insect herbivory cannot be directly predicted from caterpillar abundance or density per se, our results did not indicate, as reported in other studies (Jactel & Brockerhoff, 2007; Yguel et al., 2011), that within a native tree community, phylogenetically isolated or rare species suffer less herbivory.

### 4.3 Does phylogenetic isolation affect caterpillar richness?

According to our expectation, we found strong support for the *taxonomic isolation hypothesis*, predicting that species richness is negatively related to the phylogenetic isolation of a host plant. This negative relationship was valid for exposed feeders and shelter builders and is in line with a range of studies reporting negative relationships between phylogenetic isolation and richness for various taxonomic groups of herbivorous arthropods (Grandez‐Rios et al., 2015; Joy & Crespi, 2012; Lin et al., 2015; Vialatte et al., 2010). One explanation is that diversification in many insect phytophages has been facilitated by speciation processes following host shifts to closely related plant taxa (Drès & Mallet, 2002; Forbes et al., 2017). Host use in clades of herbivorous insects is phylogenetically constrained by a set of host plant traits (Janz & Nylin, 1998; Winkler & Mitter, 2008) that in sum make host shifts more likely among closely related plant species (Nyman et al., 2006). As a result, diverse plant lineages would be expected to harbor more lepidopteran species due to higher speciation rates than species‐poor lineages.

In addition, species richness declined with decreasing availability of the host tree species (Table 2). This supports the validity of the *species–area hypothesis*, which predicts that increasing host plant availability (i.e., leaf area) supports higher richness of associated herbivores. Several previous studies investigated the relationship between abundance of woody plants and richness of associated insect herbivores in temperate regions and found a strong positive effect of resource availability (Brändle & Brandl, 2001; Kelly & Southwood, 1999; Kennedy & Southwood, 1984; Neuvonen & Niemelä, 1981), affirming our findings.

Altogether, the patterns observed across our three study sites lead to the conclusion that species diversity of arboreal caterpillar assemblages in temperate forest ecosystems is primarily driven by those tree species that are common and have close relatives (e.g., congeners) in the plant community.

### 4.4 Does phylogenetic isolation affect specialization?

Contrary to our predictions, we observed for both guilds a negative relationship between average specialization of the caterpillar assemblages and phylogenetic isolation of the host plant tree. Thus, phylogenetically isolated tree taxa were exploited on average by less specialized species, indicating that a high fraction of nonmonophagous species were still phylogenetically constrained by utilizing closely related host plant species. The results further suggest that highly isolated plant lineages do not harbor a high percentage of monophages but were used by a substantial proportion of generalists. As stated in the methods, these results are not due to any intrinsic dependencies between phylogenetic isolation and the specialization of faunas, given DSI* is standardized by the plants available in the community and averaged across all herbivore species feeding on a given plant. Similar findings were reported by Brändle and Brandl (2001) and Vialatte et al. (2010) who found negative correlations between phylogenetic isolation of host trees and the proportion of associated specialists of herbivorous arthropods. In addition, Castagneyrol et al. (2014) reported herbivore specialists to be sensitive to host plant abundance. As we found increasing phylogenetic isolation to be accompanied by reduced foliage area, specialized Lepidoptera associated with isolated plant taxa could experience greater challenges in host detection. Moreover, focal plants might not provide sufficient resources to sustain populations of specialists in the long term. Both resource scarcity and increased difficulty in host detection could lead to temporal disappearances of some specialists and thus contribute to the observed pattern.

### 4.5 Does feeding guild matter?

Overall, assemblages of exposed feeders revealed significantly higher abundances and densities compared with those of shelter‐building caterpillars, which is contrary to findings from other studies on arboreal caterpillar communities (Diniz et al., 2012; Hrcek et al., 2013; Le Corff & Marquis, 1999). Species richness was further found to be significantly higher for exposed feeders, a pattern that was consistent among all three study sites and reported previously from our forest sites in Czech Republic (Šigut et al., 2018) and the USA (Seifert et al., 2020). The average degree of specialization was higher for shelter builders than for exposed feeders. This finding supports the assumptions that shelter builders are stronger constrained by plant defensive traits, leaf architecture, etc., compared with exposed feeders (Gaston et al., 1992; Menken et al., 2010).

Despite these general differences, our results suggested that richness, mean specialization, and assemblage composition of both exposed feeders and shelter builders are constrained in a similar way by plant phylogeny. For both guilds, compositional overlap decreased with increasing plant phylogenetic distance, while richness and mean specialization declined with increasing phylogenetic isolation of the host plant. Other studies on arthropods reported guild‐specific responses to plant phylogeny (Dinnage et al., 2012; Grandez‐Rios et al., 2015). However, guild‐specific responses to phylogenetic distance and isolation might occur only when feeding guilds forage in different environments (e.g., internal vs. external feeders) or belong to different trophic levels (e.g., herbivores vs. parasitoids).

### 4.6 Does sampling site matter?

In all our models, study site interacted with feeding guild, revealing that guilds varied regarding abundance, density, richness, and specialization among the three forests. These variations, however, are not unexpected as all three sites have different biogeographical histories and thus differ in richness of both plants and associated insect herbivores. Furthermore, herbivore species that occur in areas of higher overall richness tend to be on average more specialized than those found in species‐poor regions (Forister et al., 2015). This could directly lead to variations in specialization observed among sites. Moreover, in temperate forest ecosystems, caterpillar abundances and densities vary among years due to annual changes in climatic conditions (Reynolds et al., 2007), host plant phenology, species outbreaks (Myers & Cory, 2013), and the population vagaries of a site's natural enemy complexes. Furthermore, increased plant diversity can positively affect the intensity of top‐down control (Root, 1973). Thus, variations in plant diversity might enforce the observed differences in caterpillar abundances and densities among our temperate forest sites (see Staab & Schuldt, 2020; and references therein). Despite all these variations, our dataset revealed robust patterns that likely have general validity across many other insect–plant community types.

## 5 CONCLUSION

We demonstrated based on a large dataset of plant–caterpillar interactions from temperate forests distributed across three continents that compositional similarity, richness, and mean specialization of herbivore assemblages are structured by host plant phylogeny. Moreover, we found that differences in herbivore richness and abundance among plant taxa are affected by their commonness within the community. Our results demonstrate that both phylogenetic and compositional structures of the host plant community taken together provide promising measures to predict assemblage characteristics of its associated fauna of insect herbivores. Due to the increasing availability of phylogenetic data for plants, it is now possible to routinely employ plant phylogeny metrics to detect many of the underlying mechanisms that structure herbivore assemblages. Large‐scale studies on plant–herbivore networks, such as the one described here for temperate forests of the Northern Hemisphere, are needed to assess to what degree the patterns revealed here extend to other biomes, ecosystems, and trophic networks.

## CONFLICT OF INTEREST

The authors declare that they have no conflict of interest.

## AUTHOR CONTRIBUTION

**Carlo Lutz Seifert:** Conceptualization (lead); Data curation (equal); Formal analysis (lead); Investigation (equal); Methodology (equal); Validation (equal); Visualization (lead); Writing‐original draft (lead). **Martin Volf:** Conceptualization (supporting); Data curation (equal); Formal analysis (supporting); Investigation (equal); Methodology (equal); Project administration (equal); Validation (equal); Visualization (equal); Writing‐original draft (supporting). **Leonardo Re Jorge:** Conceptualization (equal); Formal analysis (equal); Methodology (equal); Validation (equal); Writing‐original draft (supporting). **Tomokazu Abe:** Investigation (supporting); Methodology (supporting); Validation (equal). **Grace Carscallen:** Data curation (supporting); Investigation (supporting); Validation (equal). **Pavel Drozd:** Conceptualization (equal); Investigation (equal); Methodology (equal); Project administration (lead); Validation (equal). **Rajesh Kumar:** Investigation (supporting); Methodology (supporting); Validation (equal). **Greg Lamarre:** Investigation (equal); Methodology (equal); Project administration (supporting); Validation (equal); Writing‐original draft (supporting). **Martin Libra:** Data curation (equal); Investigation (equal); Methodology (equal); Validation (equal). **Maria Eugenia Losada:** Data curation (supporting); Investigation (supporting); Validation (equal). **Scott E. Miller:** Data curation (supporting); Investigation (supporting); Methodology (supporting); Project administration (equal); Validation (equal); Writing‐original draft (supporting). **Masashi Murakami:** Investigation (equal); Methodology (equal); Project administration (lead); Validation (equal). **Geoffrey Nichols:** Data curation (supporting); Investigation (supporting); Validation (equal). **Petr Pyszko:** Data curation (equal); Investigation (equal); Methodology (equal); Validation (equal). **Martin Sigut:** Data curation (equal); Investigation (equal); Methodology (equal); Validation (equal). **David L. Wagner:** Investigation (equal); Methodology (equal); Validation (equal); Writing‐original draft (supporting). **Vojtech Novotny:** Conceptualization (equal); Funding acquisition (lead); Investigation (equal); Methodology (equal); Project administration (lead); Supervision (lead); Validation (equal); Writing‐original draft (supporting).

## Supporting information

Supplementary Material

## Data Availability

Barcode data of the sequenced caterpillar specimens included in our study are available on following databases: DS‐LANZMIK (Czech Republic; https://dx.doi.org/10.5883/DS‐LANZMIK), DS‐TOEFLEPI (Japan; https://dx.doi.org/10.5883/DS‐TOEFLEPI), and DS‐ VERTCAT (USA; https://dx.doi.org/10.5883/DS‐VERTCAT). Caterpillar–plant interaction matrices are deposited at Dryad Digital Repository (https://doi.org/10.5061/dryad.dv41ns1w6).
