# Supplementary material for: Plant phylogeny drives arboreal caterpillar assemblages across the Holarctic
Source: Ecol Evol. 2020 Nov 7;10(24):14137–51. doi: 10.1002/ece3.7005 (PMC7771119; doi:10.1002/ece3.7005)
Supplement: Supplementary file 1 — Supplementary Material [file ECE3-10-14137-s001.pdf]

Supplementary Material

Plant phylogeny drives arboreal caterpillar assemblages across the Holarctic

Carlo L. Seifert[1,2], Martin Volf[1], Leonardo R. Jorge[1,2], Tomokazu Abe[3], Grace Carscallen[4], Pavel Drozd[5], Rajesh Kumar[6], Greg P.A. Lamarre[1,2,7], Martin Libra[1,2], Maria E. Losada[4,8], Scott E. Miller[8], Masashi Murakami[3], Geoffrey Nichols[4], Petr Pyszko[5], Martin Šigut[5], David L. Wagner[9], Vojtěch Novotný[1,2]

[1] Biology Centre of the Czech Academy of Sciences, Institute of Entomology, České Budějovice, Czech Republic

[2] Faculty of Science, University of South Bohemia, České Budějovice, Czech Republic

[3] Faculty of Science, Chiba University, Chiba, Japan

[4] Conservation Ecology Center, Smithsonian Conservation Biology Institute, Front Royal, VA, USA

[5] Faculty of Science, University of Ostrava, Ostrava, Czech Republic

[6] Central Sericultural Research and Training Institute, Central Silk Board, Ministry of Textiles, Govt. of India, Pampore, Jammu and Kashmir, India

[7] ForestGEO, Smithsonian Tropical Research Institute, Balboa, Ancon, Panama

[8] National Museum of Natural History, Smithsonian Institution, Washington DC, USA

[9] University of Connecticut, Storrs, USA

*Corresponding author: Carlo L. Seifert

Biology Centre CAS; Institute of Entomology; Branisovska 1160/ 31; 37005 Ceske Budejovice; Czech Republic

Mail: carlo_seifert@web.de

Table S1 List of plant species included in this study with information about their abundance (N), leaf area, phylogenetic isolation within its community (PI), and sampling site.

Species	Family	N	Leaf area [m^2]	PI [Mya]	Site
Acer campestre L.	Sapindaceae	16	320.58	117.29	Czech Republic
Acer japonicum Thunb.	Sapindaceae	3	9.73	42.52	Japan
Acer mono Maxim.	Sapindaceae	33	457.88	46.01	Japan
Acer palmatum Thunb.	Sapindaceae	11	255.68	42.52	Japan
Acer rubrum L.	Sapindaceae	8	166.58	128.02	USA
Actinidia arguta (Siebold & Zucc.) Planch. ex. Miq.	Actinidiaceae	2	27.56	97.20	Japan
Amelanchier arborea (F.Michx.) Fernald	Rosaceae	4	38.70	66.72	USA
Carpinus betulus L.	Betulaceae	12	696.95	81.26	Czech Republic
Carpinus cordata Blume	Betulaceae	38	501.74	51.26	Japan
Carya glabra (Mill.) Sweet	Juglandaceae	15	271.86	39.84	USA
Carya tomentosa (Lam.) Nutt.	Juglandaceae	25	415.80	39.84	USA
Cercidiphyllum japonicum Siebold & Zucc. ex. J.J. Hoffm. & J.H.Schult.bis	Cercidiphyllaceae	5	50.17	136.32	Japan
Cornus controversa (Hemsl.) Soják	Cornaceae	2	16.13	121.12	Japan
Cornus florida L.	Cornaceae	2	19.20	104.22	USA
Fraxinus americana L.	Oleaceae	3	66.20	124.62	USA
Fraxinus angustifolia Vahl	Oleaceae	7	490.17	72.06	Czech Republic
Fraxinus excelsior L.	Oleaceae	2	78.20	72.06	Czech Republic
Fraxinus lanuginosa Koidz.	Oleaceae	26	60.37	69.71	Japan
Magnolia kobus DC.	Magnoliaceae	8	79.08	79.82	Japan
Magnolia obovata Thunb.	Magnoliaceae	5	70.69	79.82	Japan
Morus bombycis Koidz.	Moraceae	5	38.63	96.07	Japan
Nyssa sylvatica Marshall	Cornaceae	47	572.00	104.22	USA
Ostrya japonica Sarg.	Betulaceae	9	339.55	51.26	Japan
Ostrya virginiana (Mill.) K.Koch	Betulaceae	5	81.04	60.39	USA
Prunus avium Franch.	Rosaceae	2	10.27	55.87	USA
Prunus sargentii Rehder	Rosaceae	4	66.09	56.52	Japan
Prunus serotina Ehrh.	Rosaceae	1	19.99	55.87	USA
Prunus ssiori F.Schmidt	Rosaceae	12	119.25	56.52	Japan
Quercus alba L.	Fagaceae	10	607.83	32.48	USA
Quercus cerris L.	Fagaceae	6	229.50	55.94	Czech Republic
Quercus crispula Blume	Fagaceae	2	12.70	78.14	Japan
Quercus montana Willd.	Fagaceae	1	46.43	32.48	USA
Quercus robur L.	Fagaceae	5	450.38	55.94	Czech Republic
Quercus rubra agg.*	Fagaceae	33	1190.85	35.17	USA
Sassafras albidum (Nutt.) Nees	Lauraceae	2	26.80	149.36	USA
Sorbus alnifolia (Siebold & Zucc.) K.Koch	Rosaceae	3	15.68	67.36	Japan
Styrax obassia Siebold & Zucc.	Styracaceae	1	5.18	97.20	Japan
Syringa reticulata (Blume) H.Hara	Oleaceae	3	15.97	69.71	Japan
Tilia cordata Mill.	Malvaceae	3	71.93	117.29	Czech Republic
Tilia japonica (Miq.) Simonk.	Malvaceae	7	103.15	58.43	Japan
Tilia maximowicziana Shiras.	Malvaceae	6	132.98	58.43	Japan
Ulmus americana L.	Ulmaceae	3	52.60	95.42	USA
Ulmus laevis Pall.	Ulmaceae	5	79.77	112.30	Czech Republic

* *Quercus rubra* L. and *Quercus velutina* Lam. as well as their hybrids were pooled together as *Quercus rubra* agg.

Table S2 GenBank sequences used for the reconstruction of the host-plant phylogeny. If available, information about the respective reference publication is given.

Species	Accession Number	Authors/ Publication/ Project
**ITS**		
Acer campestre	DQ238434.1	Grimm,G.W., Renner,S.S., Stamatakis,A., Hemleben,V. (2006) "A nuclear ribosomal DNA phylogeny of Acer inferred with maximum likelihood, splits graphs, and motif analysis of 606 sequences". *Evol Bioinform Online* **2**: 279-294.
Acer japonicum	DQ238397.1	Grimm,G.W., Renner,S.S., Stamatakis,A., Hemleben,V. (2006) "A nuclear ribosomal DNA phylogeny of Acer inferred with maximum likelihood, splits graphs, and motif analysis of 606 sequences". *Evol Bioinform Online* **2**: 279-294.
Acer mono	DQ238453.1	Grimm,G.W., Renner,S.S., Stamatakis,A., Hemleben,V. (2006) "A nuclear ribosomal DNA phylogeny of Acer inferred with maximum likelihood, splits graphs, and motif analysis of 606 sequences". *Evol Bioinform Online* **2**: 279-294.
Acer palmatum	JF980312.1	Oh,D.-J., Song,G.-P., Choi,S.-A., Ko,M.-S., Yim,E.-Y., Park,S.-H., Jung,Y.-H. "Genetic analysis of plants distributed in Jeju Island" **[***GenBank project***]**
Acer rubrum	AY605460.1	Grimm,G.W., Renner,S.S., Stamatakis,A., Hemleben,V. (2006) "A nuclear ribosomal DNA phylogeny of Acer inferred with maximum likelihood, splits graphs, and motif analysis of 606 sequences". *Evol Bioinform Online* **2**: 279-294.
Actinidia arguta	AB253777.1	Taguchi,H., Watanabe,S., Hirao,T., Akiyama,H., Sakai,S., Watanabe,T., Matsuda,R., Urisu,A., Maitani,T. (2007) "Specific detection of potentially allergenic kiwifruit in foods using polymerase chain reaction" *J. Agric. Food Chem.* **55**: 1649-1655.
Carpinus betulus	HM235960.1	Lefort,F., Roemer,J., Crovadore,J. "Direct DNA amplification from plant infected tissues" **[***GenBank project***]**
Carpinus cordata	FJ011713.1	Yoo,K.-O., Wen,J. (2007) "Phylogeny of *Carpinus* and subfamily Coryloideae (Betulaceae) based on chloroplast and nuclear ribosomal sequence data." *Plant Syst. Evol.* **267:** 1-4.
Carya glabra	KF201310.1	Zhang,J.B., Li,R.Q., Xiang,X.G., Manchester,S.R., Lin,L., Wang,W., Wen,J., Chen,Z.D. (2013) "Integrated fossil and molecular data reveal the biogeographic diversification of the eastern Asian-eastern North American disjunct hickory genus (*Carya* Nutt.)". *PLoS ONE* **8**: e70449.
Carya tomentosa	EU646134.1	McCormick,M.K., Whigham,D.F., O'Neill,J.P., Becker,J.J., Werner,S., Rasmussen,H.N., Bruns,T.D., Taylor,D.L. (2009) "Abundance and distribution of *Corallorhiza odontorhiza* reflect variations in climate and ectomycorrhizae." *Ecol. Monogr.* **79**: 619-635.
Cornus controversa	JF980315.1	Oh,D.-J., Song,G.-P., Choi,S.-A., Ko,M.-S., Yim,E.-Y., Park,S.-H., Jung,Y.-H. "Genetic analysis of plants distributed in Jeju Island" **[***GenBank project***]**
Cornus florida	DQ340537.1	Xiang,Q.Y.J., Thomas,D.T., Zhang,W., Manchester,S.R., Murrell,Z. (2006) "Species level phylogeny of the genus *Cornus* (Cornaceae) based on molecular and morphological evidence--implications for taxonomy and Tertiary intercontinental migration". *Taxon* **55**:9-30.
Cercidiphyllum japonicum	AF147756.1	Zhang,Q., Shi,S., Huang,Y., Tan,F., Jin,H. and Chang,H., "The analyses of ITS sequences from Hamamelidineae and its phylogenetic significance" **[***GenBank project***]**
Fraxinus angustifolia	EU314820.1	Wallander,E. (2008) "Systematics of *Fraxinus* (Oleaceae) and evolution of dioecy". *Plant Syst. Evol.* **273**: 25-49.
Fraxinus excelsior	EU314847.1	Wallander,E. (2008) "Systematics of *Fraxinus* (Oleaceae) and evolution of dioecy". *Plant Syst. Evol.* **273**: 25-49.
Fraxinus lanuginosa	EU314857.1	Wallander,E. (2008) "Systematics of *Fraxinus* (Oleaceae) and evolution of dioecy". *Plant Syst. Evol.* **273**: 25-49.
Morus bombycis	AY345151.1	Weiguo,Z., Yile,P., Shihai,Z.Z.J.,Xuexia, M., Yongping,H. (2005) "Phylogeny of the genus *Morus* (Urticales: Moraceae) inferred from ITS and trnL-F sequences." *Afr. J. Biotechnol.* **6**: 563-569.
Ostrya japonica	FJ011754.1	Yoo,K.-O., Wen,J., "Phylogeny of subfamily Coryloideae (Betulaceae) based on seven nuclear and plastid markers" **[***GenBank project***]**
Ostrya virginiana	AF432064.1	Yoo K.-O., Wen J. (2002) "Phylogeny and biogeography of *Carpinus* and subfamily coryloideae (Betulaceae)" *Int. J. Plant Sci.* **163**:641-650.
Populus alba	JQ898650.1	Zhao,J.P., Diao,S., Zhang,B.Y., Niu,B.Q., Wang,Q.L., Wan,X.C., Luo,Y.Q. (2012). "Phylogenetic Analysis and Molecular Evolution Patterns in the MIR482-MIR1448 Polycistron of *Populus* L". *PLoS ONE* **7**: e47811.
Populus tremula	KC485108.1	Feng,J., Jiang,D., Shang,H., Zhao,C., Liu,J., Mao,K. (2013) "Barcoding poplars (*Populus* L.) from Western china". *PLoS ONE* **8:** e71710.
Prunus sargentii	AF179512.1	Lee,S., Wen,J. (2001) " A phylogenetic analysis of *Prunus* and the Amygdaloideae (Rosaceae)using ITS sequences of nuclear ribosomal DNA". *Am. J. Bot.* **88**, 150-160.
Prunus serotina	EU669104.1	Jun,W., Berggren,S.T., Lee,C.-H., Ickert-Bond,S., Yi,T.-S., Yoo,K.O., Xie,L., Shaw,J., Potter,D. (2008) "Phylogenetic inferences in *Prunus* (Rosaceae) using chloroplast ndhF and nuclear ribosomal ITS sequences." *J. Syst. Evol.* **46**: 322-332.
Quercus alba	EU646136.1	McCormick,M.K., Whigham,D.F., O'Neill,J.P., Becker,J.J., Werner,S., Rasmussen,H.N., Bruns,T.D., Taylor,D.L. (2009) "Abundance and distribution of *Corallorhiza odontorhiza* reflect variations in climate and ectomycorrhizae." *Ecol. Monogr.* **79**: 619-635.

Quercus cerris	FM243864.1	Denk,T., Grimm,G.W. (2010) "The oaks of western Eurasia: traditional classifications and evidence from two nuclear markers." *Taxon* **59**: 351-366.
Quercus montana	AY040484.1	Manos,P.S., Cannon,C.H., Zhou,Z.-K. (2001) "Systematics of Fagaceae: phylogenetic tests of reproductive trait evolution" *Int. J. Plant Sci.* **162**:1361-1379.
Quercus robur	EU628560.1	Simeone,M.C., Papini,A. "Systematic relationships of two west-Asiatic oaks, *Quercus iberica* M. Bieb. and *Quercus macranthera* Fisch. & Mey. ex Hohen., inferred from a multi-species oak molecular phylogeny" **[***GenBank project***]**
Quercus velutina	EU646142.1	McCormick,M.K., Whigham,D.F., O'Neill,J.P., Becker,J.J., Werner,S., Rasmussen,H.N., Bruns,T.D., Taylor,D.L. (2009) "Abundance and distribution of *Corallorhiza odontorhiza* reflect variations in climate and ectomycorrhizae." *Ecol. Monogr.* **79**: 619-635.
Sassafras albidum	AF272335.1	Chanderbali,A.S., van der Werff,H., Renner,S.S. (2001) "Phylogeny and historical biogeography of Lauraceae: evidence from the chloroplast and nuclear genomes" *Ann. Mo. Bot. Gard.* **88**: 104-134.
Sorbus alnifolia	FJ796908.1	Li,Q., Guo,W., Liao,W., Macklin,J.A. and Li,J. (2012) "Generic limits of Pyrinae: Insights from Sequences of Nuclear Ribosomal DNA" *Bot. Stud.* **53**: 151-164.
Styrax obassia	AF327479.1	Fritsch,P.W. (2001) "Phylogeny and biogeography of the flowering plant genus *Styrax* (Styracaceae) based on chloroplast DNA restriction sites and DNA sequences of the internal transcribed spacer region". *Mol. Phylogenet. Evol.* **19**: 387-408.
Syringa reticulata	AF297080.1	Li,J., Zhang,D., Alexander,J.H. (2001) "Classification of tree lilacs (subgenus *Ligustrina*, *Syringa*, Oleaceae): morphology and DNA sequence tell a similar story." *Harv. Pap. Bot.*517-529.
Tilia maximowicziana	KF445420.1	Melosik,I., Ciupinska,M., Winnicka,K., Koukoulas,G. (2014). "Species/geographic boundaries and evolutionary interrelationships of cultivated linden-trees (*Tilia* L.) based on morphological and nrDNA ITS characteristics". *J. Biodivers. Environ. Sci.* **5**: 90-118.
Ulmus americana	AF174640.1	Jackson,R.B., Moore,L.A., Hoffmann,W.A.,Pockman,W.T., Linder CR. (1999) "Ecosystem rooting depth determined with caves and DNA" *Proc. Natl. Acad. Sci. U.S.A.* **96**: 11387-11392.
Ulmus laevis	KC539599.1	Neubig,K.M., Herrera,F., Manchester,S.R., Germain-Aubrey,C., Abbott,J.R., Whitten,W.M. "Building the elm tree: expanded phylogenetics of Ulmaceae using DNA data, fossils, biogeography and dating" **[***GenBank project***]**

matK

Acer japonicum	AB872515.1	Nakadai,R., Murakami,M., Hirao,T. (2014) "Effects of phylogeny, leaf traits, and the altitudinal distribution of host plants on herbivore assemblages on congeneric *Acer* species". *Oecologia* **175:** 1237-1245.
Acer palmatum	AB038174.1	Kita,Y., Kato,M. "Phylogenetic relationships of the aquatic angiosperm family Podostemaceae inferred from matK sequence data" **[***GenBank project***]**
Actinidia arguta	AF322596.1	Li,J., Huang,H., Sang T. (2002) "Molecular phylogeny and infrageneric classification of *Actinidia* (Actinidiaceae)" *Syst. Bot.* **27**: 408-415.
Carpinus cordata	AY211986.1	Yoo,K.-O., Wen,J., "Phylogeny of subfamily Coryloideae (Betulaceae) based on seven nuclear and plastid markers" **[***GenBank project***]**
Carya glabra	KP402302.1	Lemoine, Nathan P. et al. "No difference in assembly mechanisms for native and introduced species." **[***GenBank project***]**
Carya tomentosa	AF118039.1	Stanford AM., Harden R., Parks CR. (2000) "Phylogeny and biogeography of *Juglans* (Juglandaceae) based on matK and ITS sequence data" *Am. J. Bot.* **87**: 872-882.
Cercidiphyllum japonicum	AB490219.1	Kokubun,H., Watanabe,H., Koizumi,M., Hashida,H. and Ando,T. "Intraspecific phylogeographical structure reflecting a Tertiary event" **[***GenBank project***]**
Cornus controversa	U96893.1	Xiang,Q., Soltis,D., Soltis,P. (1998) "Phylogenetic relationships of Cornaceae and close relatives inferred from matK and rbcL sequences". *Am. J. Bot.* **85**: 285.
Cornus florida	EU002175.1	Wang,H., Moore, M.J., Soltis,P.S., Bell,C.D., Brockington,S.F., Alexandre,R., Davis,C.C., Latvis,M., Manchester,S.R., Soltis,D.E. (2009) "Rosid radiation and the rapid rise of angiosperm-dominated forests" *Proc. Natl. Acad. Sci. U.S.A.* **106**: 3853-3858.
Fraxinus americana	HQ593299.1	Burgess,K.S., Fazekas,A.J., Kesanakurti,P.R., Graham,S.W., Husband,B.C., Newmaster,S.G., Percy,D.M., Hajibabaei,M., Barrett,S.C.H. (2011) "Discriminating plant species in a local temperate flora using the rbcL+ matK DNA barcode." *Methods Ecol. Evol.* **2**: 333-340.
Fraxinus excelsior	AM933427.1	Besnard,G., de Cesas,R.B., Christin,P.-A., Vargas,P. (2009) "Phylogenetics of *Olea* (Oleaceae) based on plastid and nuclear ribosomal DNA sequences: tertiary climatic shifts and lineage differentiation times." *Ann. Bot.* **104**: 143-160.
Magnolia kobus	JX280396.1	Song,E., Kim,S. "Phylogeny of Magnoliaceae based on cp genomes" **[***GenBank project***]**
Morus bombycis	GU145560.1	Venkateswarlu,M., Ravikumar,G., Nair,V.C. "Testing candidate plant barcode regions in 13 *Morus* species" **[***GenBank project***]**
Nyssa sylvatica	GU266599.1	Hilu, K.W., Black, C., Diouf, D., Burleigh, J.G. (2008) "Phylogenetic signal in matK vs. trnK: a case study in early diverging eudicots (angiosperms)" *Mol. Phylogenet. Evol.* **48**: 1120-1130.
Ostrya japonica	AY212005.1	Yoo,K.-O., Wen,J., "Phylogeny of subfamily Coryloideae (Betulaceae) based on seven nuclear and plastid markers" **[***GenBank project***]**
Ostrya virginiana	AY212009.1	Yoo,K.-O., Wen,J. (2007) "Phylogeny of *Carpinus* and subfamily Coryloideae (Betulaceae) based on chloroplast and nuclear ribosomal sequence data." *Pl Syst. Evol.* **267**: 25-35.

Prunus avium	JN895117.1	de Vere,N., Rich,T.C.G., Ford,C.R., Trinder,S.A., Long,C., Moore,C.M., Satterthwaite,D., Davies,H., Allainguillaume,J., Ronca,S., Tatarinova,T., Garbett,H., Walker,K., Wilkinson,M.J. "Creation of a DNA database for a nation's native flowering plants and conifers" **[***GenBank project***]**
Prunus sargentii	KF154805.1	Shi,S., Li,J., Sun,J., Yu,J., Zhou,S. (2013) "Phylogeny and Classification of *Prunus* sensu lato (Rosaceae)". *J. Integr. Plant. Biol.* **55**, 1069-1079.
Prunus serotina	HQ235267.1	Cohen I.M., Shaw J., Wen J., Potter D. "Plant DNA Barcoding: Testing the Utility of Two 'Agreed' Upon Loci" **[***GenBank project***]**
Quercus alba	EU749360.1	Fazekas, A.J., Burgess,K.S., Kesanakurti,P.R., Graham,S.W., Newmaster,S.G., Husband,B.C., Percy,D.M., Hajibabaei,M., Barrett,S.C. (2008) "Multiple multilocus DNA barcodes from the plastid genome discriminate plant species equally well" *PLoS ONE* **3**: e2802.
Quercus crispula	AB727873.1	Liu, H.-Z., Harada,K. (2014) "Geographic distribution and origin of the chloroplast T/C-type in *Quercus mongolica* var. *crispula* in northeastern Japan." *Plant Species Biol.* **29**: 207-211.
Quercus montana	KR062091.1	Sundaram,M., Willoughby,J.R., Lichti,N.I., Steele,M.A., Swihart,R.K. (2015) "Segregating the effects of seed traits and common ancestry of hardwood trees on eastern gray squirrel foraging decisions" *PLoS ONE* **10**: e0130942.
Quercus rubra	U92864.1	Manos,P., Steele,K. (1997) "Phylogenetic analyses of "higher" Hamamelididae based on plastid sequence data" *Am. J. Bot.* **84**: 1407.
Quercus velutina	KJ593084.1	Wolf,A., Howe,R., Parker,J., Erickson,D., Kress,J. "Differential distributions of community phylogenetic diversity among life history forms in tropical and temperate plant species" **[***GenBank project***]**
Sassafras albidum	EU153879.1	Madrinan S., Chacon J. "DNA Barcodes of Lauraceae" **[***GenBank project***]**
Sorbus alnifolia	DQ860451.1	Campbell,C.S., Evans,R.C., Morgan,D.R., Dickinson,T.A., Arsenault,M.P. (2007) "Phylogeny of subtribe Pyrinae (formerly the Maloideae, Rosaceae): limited resolution of a complex evolutionary history". *Plant Syst. Evol.* **266**: 119-145.
Syringa reticulata	JN590998.1	Li,J., Goldman-Huertas,B., DeYoung,J., Alexander III,J. (2012) "Phylogenetics and diversification of *Syringa* inferred from nuclear and plastid DNA sequences." *Castanea* **77***:* 82-88.
Ulmus americana	KC539607.1	Neubig,K.M., Herrera,F., Manchester,S.R., Germain-Aubrey,C., Abbott,J.R., Whitten,W.M. "Building the elm tree: expanded phylogenetics of Ulmaceae using DNA data, fossils, biogeography and dating" **[***GenBank project***]**

rbcL

Acer campestre	DQ978399.1	Renner,S.S., Beenken,L., Grimm,G.W., Kocyan,A., Ricklefs,R.E. (2007) "The evolution of dioecy, heterodichogamy, and labile sex expression in *Acer*". *Evolution* **61**: 2701-2719.
Acer japonicum	AB872548.1	Nakadai,R., Murakami,M., Hirao,T. (2014) "Effects of phylogeny, leaf traits, and the altitudinal distribution of host plants on herbivore assemblages on congeneric *Acer* species". *Oecologia* **175**, 1237-1245.
Acer mono	DQ978416.1	Renner,S.S., Beenken,L., Grimm,G.W., Kocyan,A., Ricklefs,R.E. (2007) "The evolution of dioecy, heterodichogamy, and labile sex expression in *Acer*". *Evolution* **61**: 2701-2719.
Acer palmatum	DQ978421.1	Renner,S.S., Beenken,L., Grimm,G.W., Kocyan,A., Ricklefs,R.E. (2007) "The evolution of dioecy, heterodichogamy, and labile sex expression in *Acer*". *Evolution* **61**: 2701-2719.
Acer rubrum	DQ978428.1	Renner,S.S., Beenken,L., Grimm,G.W., Kocyan,A., Ricklefs,R.E. (2007) "The evolution of dioecy, heterodichogamy, and labile sex expression in *Acer*". *Evolution* **61**: 2701-2719.
Actinidia arguta	AJ549049.1	Chat,J., Jáuregui,B., Petit,R.J., Nadot, S. (2004) "Reticulate evolution in kiwifruit (*Actinidia*, Actinidiaceae) identified by comparing their maternal and paternal phylogenies" *Am. J. Bot.* **91**: 736-747.
Amelanchier arborea	JQ391253.1	Lo, E.Y., Donoghue,M.J. (2012) "Expanded phylogenetic and dating analyses of the apples and their relatives (*Pyreae*, Rosaceae)" *Mol. Phylogenet. Evol.* **63**: 230-243.
Carpinus betulus	AY263928.1	Li,R.-Q., Chen,Z.-D., Lu,A.-M., Soltis,D.E., Soltis,P.S., Manos,P.S. (2004) "Phylogenetic relationships in Fagales based on DNA sequences from three genomes." *Int. J. Plant Sci.* **165**: 311-324.
Carpinus cordata	KF418945.1	Xiang,X.-G., Wang,W., Li,R.-Q., Lin,L., Liu,Y., Zhou,Z.-K., Li,Z.-Y., Chen,Z.-D. (2014) "The interplay of diaspores and environments triggers increased diversification of Fagales in the Paleogene". *Perspect. Plant Ecol. Evol. Syst.* **16**, 101-110.
Carya glabra	AF119186.1	Wang,M., Lincoln,D.E. "Origin of volatile terpenoids inferred from rbcL gene phylogeny" **[***GenBank project***]**
Carya tomentosa	KP644076.1	Erickson,D., Kuzmina,M., Kress,J., McShea,W. "Forensic analysis of white tailed deer diet" **[***GenBank project***]**
Cercidiphyllum japonicum	L11673.1	Olmstead,R.G., Michaels,H.J., Scott,K.M., Palmer,J.D. (1992) "Monophyly of the Asteridae and identification of their major lineages inferred from DNA sequences of rbcL". *Ann. Mo. Bot. Gard.* **79**, 249-265.
Cornus controversa	AF190433.1	Xiang,Q.-Y., Soltis,D.E., Soltis,P.S., Manchester,S.R., Crawford,D.J. (2000) "Timing the Eastern Asian-Eastern North American floristic disjunction: molecular clocks corroborates paleontological estimates". *Mol. Phylogenet. Evol.* **15**: 462-472.
Cornus florida	EU002276.1	Wang,H., Moore,M.J., Soltis,P.S., Bell,C.D., Brockington,S.F., Alexandre,R., Davis,C.C., Latvis,M., Manchester,S.R., Soltis,D.E. (2009) "Rosid radiation and the rapid rise of angiosperm-dominated forests" *Proc. Natl. Acad. Sci. U.S.A.* **106**: 3853-3858.
Fraxinus americana	KJ773520.1	Germain-Aubrey,C.C., Allen,J.M., Neubig,K.M., Abbott,J.R., Majure,L.C., Guralnick,R., Soltis,D.E., Soltis,P.S. "Phylogenetic diversity of Florida plants" **[***GenBank project***]**

Fraxinus angustifolia	HG765055.1	Laiou,A., Mandolini,L.A., Piredda,R., Bellarosa,R., Simeone,M.C. (2013) "DNA barcoding as a complementary tool for conservation and valorisation of forest resources". *Zookeys* **365**: 197-213.
Fraxinus excelsior	FJ395592.1	James,K.E., Rumsey,F., Spencer,M., Carine,M., Vogel,J.C., Schneider,H. "Barcoding Darwin's meadow: high-throughput DNA barcoding from specimen to sequence". **[***GenBank project***]**
Magnolia kobus	AY743438.1	Pirie,M.D., Chatrou,L.W., Erkens,R.H.J., Maas,J.W., van der Niet,T., Mols,J.B., Richardson,J.E. (2005) "Phylogeny reconstruction and molecular dating in four Neotropical genera of Annonaceae: the effect of taxon sampling in age estimations". *Regnum Vegetabile* **143**: 149-174.
Nyssa sylvatica	AF119178.1	Wang,M., Lincoln,D.E. "Origin of volatile terpenoids inferred from rbcL gene phylogeny" **[***GenBank project***]**
Ostrya japonica	KF418957.1	Xiang,X.-G., Wang,W., Li,R.-Q., Lin,L., Liu,Y., Zhou,Z.-K., Li,Z.-Y., Chen,Z.-D. (2014) "The interplay of diaspores and environments triggers increased diversification of Fagales in the Paleogene". *Perspect. Plant Ecol. Evol. Syst.* **16**, 101-110.
Ostrya virginiana	X56620.1	Bousquet,J., Strauss,S.H., Li,P. "Complete congruence between morphological and rbcL-based molecular phylogenies in birches and related species (Betulaceae)" *Mol. Biol. Evol*. **9**: 1076-1088.
Populus alba	HM850277.1	Schaefer,H., Hardy,O.J., Silva,L., Barraclough,T.G., Savolainen,V. (2011) "Testing Darwin's naturalization hypothesis in the Azores" . *Ecol. Lett.* **14**, 389-396.
Populus tremula	AJ418827.1	Chase,M.W., Zmarzty,S., Lledo,M.D., Wurdack,K.J., Swensen,S.M.Fay,M.F. (2002) "When in doubt, put it in Flacourtiaceae: a molecular phylogenetic analysis based on plastid rbcL DNA sequences." *KEW Bull.* **57**: 141-181.
Prunus avium	HQ235394.1	Cohen,I.M., Shaw,J., Wen,J., Potter,D. "Plant DNA barcoding: testing the utility of two 'agreed' upon loci" **[***GenBank project***]**
Prunus sargentii	AY052515.1	Jung,Y.H., Han,S.H., Oh,Y.S., Oh,M.Y. "*Prunus sargentii* (34p4p3) sequence of rbcL gene" **[***GenBank project***]**
Prunus serotina	HQ590225.1	Burgess,K.S., Fazekas,A.J., Kesanakurti,P.R., Graham,S.W., Husband,B.C., Newmaster,S.G., Percy,D.M., Hajibabaei,M., Barrett,S.C.H. (2011) "Discriminating plant species in a local temperate flora using the rbcL+ matK DNA barcode." *Methods Ecol. Evol.* **2**: 333-340.
Quercus alba	KF418899.1	Xiang,X.-G., Wang,W., Li,R.-Q., Lin,L., Liu,Y., Zhou,Z.-K., Li,Z.-Y., Chen,Z.-D. (2014) "The interplay of diaspores and environments triggers increased diversification of Fagales in the Paleogene" *Perspect. Plant Ecol. Evol. Syst.* **16**: 101-110.
Quercus cerris	AB125017.1	Kamiya,K., Harada,K., Ogino,K., Clyde,M.M., Latiff,A.M. (2003) "Phylogeny and genetic variation of Fagaceae in tropical montane forests." *Tropics* **13**: 119-125.
Quercus crispula	MF044861.1	Hermida-Carrera,C., Fares,M.A., Fernández,Á., Gil-Pelegrín,E., Kapralov,M.V., Mir,A., Molins,A., Peguero-Pina,J.J., Rocha,J., Sancho-Knapik,D., Galmés,J. (2017) "Positively selected amino acid replacements within the RuBisCO enzyme of oak trees are associated with ecological adaptations" *PLoS ONE* **12**: e0183970.
Quercus montana	MF044862.1	Hermida-Carrera,C., Fares,M.A., Fernández,Á., Gil-Pelegrín,E., Kapralov,M.V., Mir,A., Molins,A., Peguero-Pina,J.J., Rocha,J., Sancho-Knapik,D., Galmés,J. (2017) "Positively selected amino acid replacements within the RuBisCO enzyme of oak trees are associated with ecological adaptations" *PLoS ONE* **12**: e0183970.
Quercus robur	AB125025.1	Kamiya,K., Harada,K., Ogino,K., Clyde,M.M., Latiff,A.M. (2003) "Phylogeny and genetic variation of Fagaceae in tropical montane forests." *Tropics* **13**: 119-125.
Quercus rubra	M58391.1	Bousquet,J., Strauss,S.H., Li,P. (1992) "Complete congruence between morphological and rbcL-based molecular phylogenies in birches and related species (Betulaceae)" *Mol. Biol. Evol*. **9**: 1076-1088.
Quercus velutina	MF044938.1	Hermida-Carrera,C., Fares,M.A., Fernández,Á., Gil-Pelegrín,E., Kapralov,M.V., Mir,A., Molins,A., Peguero-Pina,J.J., Rocha,J., Sancho-Knapik,D., Galmés,J. (2017) "Positively selected amino acid replacements within the RuBisCO enzyme of oak trees are associated with ecological adaptations" *PLoS ONE* **12**: e0183970.
Sassafras albidum	AF206819.1	Soltis,P.S., Soltis,D.E., Chase,M.W. (1999) "Angiosperm phylogeny inferred from multiple genes as a tool for comparative biology." *Nature* **402**: 402-404.
Styrax obassia	AF396158.1	Fritsch,P.W., Morton,C.M., Chen,T., Meldrum,C. (2001) "Phylogeny and biogeography of the Styracaceae." *Int. J. Plant Sci.* **162**: S95-S116.
Tilia cordata	KP088885.1	Dong,W., Xu,C., Li,C., Sun,J., Zuo,Y., Shi,S., Cheng,T., Guo,J., Zhou,S. (2015) "Ycf1, the most promising plastid DNA barcode of land plants". *Sci. Rep.* **5**, 8348.
Ulmus americana	AF500337.1	Sytsma,K.J., Morawetz,J., Pires,J.C., Nepokroeff,M., Conti,E., Zjhra,M., Hall,J.C., Chase,M.W. (2002) "Urticalean rosids: circumscription, rosid ancestry, and phylogenetics based on rbcL, trnL-F, and ndhF sequences" *Am. J. Bot.* **89**: 1531-154.
Ulmus laevis	KC539702.1	Neubig,K.M., Herrera,F., Manchester,S.R., Germain-Aubrey,C., Abbott,J.R., Whitten,W.M. "Building the elm tree: expanded phylogenetics of Ulmaceae using DNA data, fossils, biogeography and dating". **[***GenBank project***]**

trnL-trnF

Acer campestre	AF401189.1	Tian,X., Guo, Z.H.,Li,D.Z. (2002) "Phylogeny of Aceraceae based on ITS and trnL-F data sets." *Acta Bot. Sin.* **44**: 714-724.
Acer japonicum	AJ413167.1	Pfosser,M.F., Guzy-Wrobelska,J., Sun,B.Y., Stuessy,T.F.,Sugawara,T., Fujii,N. (2002) "The origin of species of *Acer* (Sapindaceae) endemic to Ullung Island, Korea". *Syst. Bot.* **27**, 351-367.
Acer mono	JN102147.1	Oh,D.-J., Song,G.-P., Choi,S.-A., Ko,M.-S., Yim,E.-Y., Park,S.-H., Jung,Y.-H. "Genetic analysis of plants distributed in Jeju Island" **[***GenBank project***]**
Acer palmatum	AJ413165.1	Pfosser,M.F., Guzy-Wrobelska,J., Sun,B.Y., Stuessy,T.F.,Sugawara,T., Fujii,N. (2002) "The origin of species of *Acer* (Sapindaceae) endemic to Ullung Island, Korea". *Syst. Bot.* **27**, 351-367.

Actinidia arguta	AY156914.1	Jung,Y., Kim,K., Kang,S., Jun,S., Oh,M., Kim,S. (2003) “Phylogenetic analysis of the genus Actinidia in Korea inferred from two non-coding chloroplast DNA sequences” *Hanguk Yujon Hakhoe Chi* **25**: 95-101.
Carpinus betulus	FJ012011.1	Yoo,K.-O., Wen,J., "Phylogeny of subfamily Coryloideae (Betulaceae) based on seven nuclear and plastid markers". **[***GenBank project***]**
Carpinus cordata	AY211400.1	Yoo,K.-O., Wen,J., "Phylogeny of subfamily Coryloideae (Betulaceae) based on seven nuclear and plastid markers". **[***GenBank project***]**
Carya glabra	AF303796.1	Manos P.S., Stone D.E. (2001) “Evolution, phylogeny, and systematics of the Juglandaceae” *Ann. Mo. Bot. Gard.* **88**: 231-269.
Carya tomentosa	AF303797.1	Manos P.S., Stone D.E. (2001) “Evolution, phylogeny, and systematics of the Juglandaceae” *Ann. Mo. Bot. Gard.* **88**: 231-269.
Cercidiphyllum japonicum	AM397171.1	Worberg,A., Quandt,D., Barniske,A.M., Loehne,C., Hilu,K.W., Borsch,T. (2007) "Phylogeny of basal eudicots: Insights from non-coding and rapidly evolving DNA". *Org. Divers. Evol*. **7**: 55-77.
Cornus florida	AY254238.1	Hufford, L., McMahon,M.M., Sherwood,A.M., Reeves,G., Chase,M.W. (2003) “The major clades of Loasaceae: phylogenetic analysis using the plastid matK and trnL-trnF regions” *Am. J. Bot.* **90**: 1215-1228.
Fraxinus americana	AF231825.1	Wallander, E., Albert,V.A. (2000) “Phylogeny and classification of Oleaceae based on rps16 and trnL-F sequence data” *Am. J. Bot.* **87**: 1827-1841.
Fraxinus excelsior	AY911646.1	Harbourne,M.E., Douglas,G.C., Waldren,S., Hodkinson,T.R. (2005) "Characterization and primer development for amplification of chloroplast microsatellite regions of Fraxinus excelsior". *J. Plant Res.* **118**, 339-341.
Magnolia kobus	AY743457.1	Pirie,M.D., Chatrou,L.W., Erkens,R.H.J., Maas,J.W., van der Niet,T., Mols,J.B., Richardson,J.E. (2005) "Phylogeny reconstruction and molecular dating in four Neotropical genera of Annonaceae: the effect of taxon sampling in age estimations". *Regnum Vegetabile* **143**: 149-174.
Magnolia obovata	AB570261.1	Wu,Y., Zhu,S., Komatsu,K. "Identification of herbal drugs by DNA sequences". **[***GenBank project***]**
Morus bombycis	JN006417.1	Chen,R.-F., Yu,M.-D., Zhang,Z., Xu,L., Wang,X.-L., Tang,Z. (2011) "Morus ITS, trnL-F, rps16 sequence and phylogenetic analysis of mulberry resources". *Zhongguo Nong Ye Ke Xue* **44**: 1553-1561.
Nyssa sylvatica	JF321161.1	Xiang,Q.Y., Thomas,D.T., Xiang,Q.P. (2011) “Resolving and dating the phylogeny of Cornales-effects of taxon sampling, data partitions, and fossil calibrations” *Mol. Phylogenet. Evol.* **59**: 123-138.
Ostrya japonica	AY211421.1	Yoo,K.-O., Wen,J., "Phylogeny of subfamily Coryloideae (Betulaceae) based on seven nuclear and plastid markers". **[***GenBank project***]**
Prunus avium	FJ899123.1	Davies C.J.J., Milne R.I., Sales F. “Species relationships of *Prunus* subgenus Laurocerasus and allies” **[***GenBank project***]**
Prunus sargentii	AF429919.1	Jung,Y.H., Han,S.H., Oh,Y.S. and Oh,M.Y. **[***GenBank project***]**
Prunus serotina	JX414453.1	Chin,S.-W., Potter,D. “Early establishment and strong resilience: evolutionary history and phylogeography of desert almond, Prunus fasciculata (Rosaceae) in Mojave, Sonoran and Great Basin Deserts” **[***GenBank project***]**
Quercus alba	KF419044.1	Xiang,X.-G., Wang,W., Li,R.-Q., Lin,L., Liu,Y., Zhou,Z.-K., Li,Z.-Y., Chen,Z.-D. (2014) “The interplay of diaspores and environments triggers increased diversification of Fagales in the Paleogene” *Perspect. Plant Ecol. Evol. Syst.* **16**:101-110.
Quercus cerris	HM770073.1	Paule,J., Newbury,H.John., Ford-Lloyd,B.V. "CpDNA variation in Carpathian oaks (*Quercus* L.)" **[***GenBank project***]**
Quercus crispula	AB727893.1	Liu, H.-Z., Harada,K. (2014) "Geographic distribution and origin of the chloroplast T/C-type in *Quercus mongolica* var. *crispula* in northeastern Japan." *Plant Species Biol.* **29**: 207-211.
Quercus robur	HM770066.1	Paule,J., Newbury,H.John., Ford-Lloyd,B.V. "CpDNA variation in Carpathian oaks (*Quercus* L.)" **[***GenBank project***]**
Quercus rubra	FJ490789.1	Ferri,G., Alú,M., Corradini,B., Beduschi,G. (2009) "Forensic botany: species identification of botanical trace evidence using a multigene barcoding approach." *Int. J Legal Med.* **123**: 395-401.
Sassafras albidum	KF586681.1	Massoni,J., Forest,F., Sauquet,H. (2014) “Increased sampling of both genes and taxa improves resolution of phylogenetic relationships within Magnoliidae, a large and early-diverging clade of angiosperms” *Mol. Phylogenet. Evol.* **70**: 84-93.
Sorbus alnifolia	DQ863223.1	Campbell,C.S., Evans,R.C., Morgan,D.R., Dickinson,T.A., Arsenault,M.P. (2007) "Phylogeny of subtribe Pyrinae (formerly the Maloideae, Rosaceae): limited resolution of a complex evolutionary history". *Plant Syst. Evol.* **266**: 119-145.
Styrax obassia	AB237440.1	Iwasaki,T., Aoki,K., Seo,A., Murakami,N. (2006) "Intraspecific sequence variation of chloroplast DNA among the component species of deciduous broad-leaved forests in Japan". *J. Plant Res.* **119**: 539-552.
Syringa reticulata	JN591017.1	Li,J., Goldman-Huertas,B., DeYoung,J., Alexander III,J. (2012) "Phylogenetics and diversification of *Syringa* inferred from nuclear and plastid DNA sequences." *Castanea* ***77:*** 82-88.
Ulmus americana	KC539710.1	Neubig,K.M., Herrera,F., Manchester,S.R., Germain-Aubrey,C., Abbott,J.R., Whitten,W.M. “Building the elm tree: expanded phylogenetics of Ulmaceae using DNA data, fossils, biogeography and dating” **[***GenBank project***]**
Ulmus laevis	KC539736.1	Neubig,K.M., Herrera,F., Manchester,S.R., Germain-Aubrey,C., Abbott,J.R., Whitten,W.M. "Building the elm tree: expanded phylogenetics of Ulmaceae using DNA data, fossils, biogeography and dating". **[***GenBank project***]**

Table S3 List of caterpillar species included in this study with information about feeding guild (Exp = exposed feeders; She = shelter builders), specialisation (measured as distance-based specialisation index, DSI*), abundance (N), and sampling site.

Family	Species name	Guild	DSI*	N	Site
Batrachedridae	*Batrachedra* sp. 1	She	1,00	5	Japan
Bombycidae	*Oberthueria falcinegra*	Exp	0,95	4	Japan
Brahmaeidae	*Brahmaea japonica*	Exp	0,64	8	Japan
Bucculatricidae	*Bucculatrix packardella*	Exp	0,05	4	USA
Bucculatricidae	*Bucculatrix* sp. ADL1829	Exp	1,00	4	USA
Bucculatricidae	*Bucculatrix thoracella*	Exp	0,97	566	Czech Republic
Bucculatricidae	*Bucculatrix ulmella*	Exp	0,72	36	Czech Republic
Chimabachidae	*Dasystoma salicella*	She	0,72	10	Czech Republic
Chimabachidae	*Diurnea fagella*	She	0,02	52	Czech Republic
Chimabachidae	*Diurnea lipsiella*	She	0,02	12	Czech Republic
Coleophoridae	*Coleophora flavipennella*	She	0,83	27	Czech Republic
Coleophoridae	*Coleophora kuehnella*	She	0,96	4	Czech Republic
Crambidae	*Palpita magniferalis*	She	1,00	15	USA
Depressariidae	*Antaeotricha schlaegeri*	She	0,95	8	USA
Depressariidae	*Machimia tentoriferella*	She	-0,22	483	USA
Depressariidae	*Odites leucostola*	She	0,69	26	Japan
Depressariidae	*Psilocorsis quercicella*	She	0,96	28	USA
Depressariidae	*Psilocorsis reflexella*	She	0,96	768	USA
Depressariidae	*Rectiostoma xanthobasis*	She	0,97	12	USA
Depressariidae	*Semioscopis merriccella*	She	1,00	12	USA
Depressariidae	*Semioscopis packardella*	She	1,00	4	USA
Drepanidae	*Auzata superba*	She	1,00	3	Japan
Drepanidae	*Watsonalla binaria*	Exp	0,48	8	Czech Republic
Erebidae	*Allotria elonympha*	Exp	1,00	28	USA
Erebidae	*Arctornis l nigrum*	Exp	0,37	5	Japan
Erebidae	*Calliteara pudibunda*	Exp	0,70	11	Czech Republic
Erebidae	*Catocala lara*	Exp	1,00	22	Japan
Erebidae	*Catocala sponsa*	Exp	0,75	15	Czech Republic
Erebidae	*Dasychira obliquata*	Exp	-0,13	8	USA
Erebidae	*Dasychira tephra*	Exp	0,53	18	USA
Erebidae	*Euproctis piperita*	Exp	0,35	4	Japan
Erebidae	*Euproctis similis*	Exp	-0,12	13	Czech Republic
Erebidae	*Halysidota tessellaris*	Exp	0,38	66	USA
Erebidae	*Hypena baltimoralis*	Exp	1,00	7	USA
Erebidae	*Hyperstrotia nana*	Exp	0,69	15	USA
Erebidae	*Hyperstrotia secta*	Exp	0,46	4	USA
Erebidae	*Ivela auripes*	Exp	0,89	26	Japan
Erebidae	*Laspeyria flexula*	Exp	0,10	5	Czech Republic
Erebidae	*Lemyra infernalis*	Exp	-0,67	7	Japan
Erebidae	*Lymantria dispar*	Exp	0,52	127	Czech Republic
Erebidae	*Lymantria dispar*	Exp	0,07	50	Japan
Erebidae	*Lymantria dispar*	Exp	0,37	37	USA
Erebidae	*Lymantria mathura*	Exp	0,19	2867	Japan
Erebidae	*Lymantria monacha*	Exp	1	4	Czech Republic

Erebidae	*Lymantria monacha*	Exp	0,35	9	Japan
Erebidae	*Orgyia antiqua*	Exp	0,18	34	Czech Republic
Erebidae	*Orgyia definita*	Exp	0,44	32	USA
Erebidae	*Orgyia leucostigma*	Exp	-0,13	24	USA
Erebidae	*Orgyia recens*	Exp	1	179	Czech Republic
Erebidae	*Panopoda carneicosta*	Exp	0,23	5	USA
Erebidae	*Panopoda rufimargo*	Exp	0,98	14	USA
Erebidae	*Parallelia bistriaris*	Exp	0,70	22	USA
Erebidae	*Sypnoides hercules*	Exp	0,63	36	Japan
Gelechiidae	*Anarsia bimaculata*	She	1,00	4	Japan
Gelechiidae	*Aristotelia* sp. AAH5604	She	1,00	3	USA
Gelechiidae	*Arogalea cristifasciella*	She	0,53	9	USA
Gelechiidae	*Chionodes adamas*	She	0,96	38	USA
Gelechiidae	*Chionodes bicostomaculella*	She	0,97	37	USA
Gelechiidae	*Chionodes fuscomaculella*	She	0,96	22	USA
Gelechiidae	*Chionodes pereyra*	She	0,90	44	USA
Gelechiidae	*Cnaphostola biformis*	She	0,71	9	Japan
Gelechiidae	*Coleotechnites quercivorella*	She	1,00	4	USA
Gelechiidae	*Dichomeris georgiella*	She	0,70	18	USA
Gelechiidae	*Dichomeris ligulella*	She	0,95	3	USA
Gelechiidae	*Dichomeris ustalella*	She	0,20	7	Czech Republic
Gelechiidae	*Dichomeris ventrellus*	She	0,94	3	USA
Gelechiidae	*Faristenia geminisignella*	She	0,99	494	Japan
Gelechiidae	*Faristenia* sp. 1	She	1,00	6	Japan
Gelechiidae	*Faristenia* sp. 2	She	0,27	6	Japan
Gelechiidae	*Faristenia ussuriella*	She	1,00	3	Japan
Gelechiidae	*Polyhymno indistincta*	She	1,00	7	Japan
Gelechiidae	*Pseudotelphusa quercinigracella*	She	0,96	8	USA
Gelechiidae	*Pseudotelphusa querciphaga*	She	1,00	3	USA
Gelechiidae	*Psoricoptera gibbosella*	She	0,96	4	Czech Republic
Gelechiidae	*Recurvaria comprobata*	She	0,31	4	Japan
Gelechiidae	*Trypanisma prudens*	She	1,00	3	USA
Geometridae	*Acrodontis kotshubeji*	Exp	0,10	5	Japan
Geometridae	*Agaraeus parva*	Exp	-0,45	12	Japan
Geometridae	*Agriopis aurantiaria*	Exp	0,45	243	Czech Republic
Geometridae	*Agriopis leucophaearia*	Exp	0,95	238	Czech Republic
Geometridae	*Agriopis marginaria*	Exp	0,16	158	Czech Republic
Geometridae	*Alsophila aceraria*	Exp	0,95	4	Czech Republic
Geometridae	*Alsophila aescularia*	Exp	-0,37	108	Czech Republic
Geometridae	*Alsophila japonensis*	Exp	0,00	16	Japan
Geometridae	*Alsophila pometaria*	Exp	0,21	24	USA
Geometridae	*Apocheima hispidaria*	Exp	0,66	6	Czech Republic
Geometridae	*Asthena albulata*	Exp	1	6	Czech Republic
Geometridae	*Besma quercivoraria*	Exp	0,95	6	USA
Geometridae	*Calleulype whitelyi*	Exp	0,20	243	Japan
Geometridae	*Campaea margaritaria*	Exp	-0,31	82	Czech Republic
Geometridae	*Campaea perlata*	Exp	-0,14	3	USA
Geometridae	*Chloroclysta siterata*	Exp	0,24	19	Czech Republic
Geometridae	*Cleora sublunaria*	Exp	-0,14	12	USA
Geometridae	*Colotois pennaria*	Exp	0,40	178	Czech Republic

Geometridae	*Cryptochorina amphidasyaria*	Exp	0,07	5	Japan
Geometridae	*Cyclophora annularia*	Exp	1	101	Czech Republic
Geometridae	*Cyclophora punctaria*	Exp	0,96	8	Czech Republic
Geometridae	*Cyclophora ruficiliaria*	Exp	0,93	42	Czech Republic
Geometridae	*Ectropis crepuscularia*	Exp	0,04	27	Czech Republic
Geometridae	*Ennomos autumnaria*	Exp	-0,53	5	Czech Republic
Geometridae	*Ennomos fuscantaria*	Exp	0,99	14	Czech Republic
Geometridae	*Ennomos nephotropa*	Exp	0,52	10	Japan
Geometridae	*Ennomos quercinaria*	Exp	0,68	7	Czech Republic
Geometridae	*Epirrita dilutata*	Exp	0,71	12	Czech Republic
Geometridae	*Epirrita viridipurpurescens*	Exp	0,03	263	Japan
Geometridae	*Erannis defoliaria*	Exp	0,43	282	Czech Republic
Geometridae	*Erannis defoliaria*	Exp	0,00	24	Japan
Geometridae	*Erannis golda*	Exp	0,31	443	Japan
Geometridae	*Eulithis convergenata*	Exp	0,39	67	Japan
Geometridae	*Eupithecia abbreviata*	Exp	-0,31	6	Czech Republic
Geometridae	*Eupithecia fletcherana*	Exp	1,00	3	USA
Geometridae	*Eupithecia jejunata*	Exp	-0,28	3	USA
Geometridae	*Eupithecia* sp. 1	Exp	0,33	16	Japan
Geometridae	*Eupithecia* sp. 2	Exp	0,12	47	Japan
Geometridae	*Eupithecia swettii*	Exp	0,33	20	USA
Geometridae	*Eutrapela clemataria*	Exp	-0,46	14	USA
Geometridae	*Gandaritis festinaria*	Exp	0,17	20	Japan
Geometridae	*Garaeus specularis*	Exp	-0,35	24	Japan
Geometridae	*Geometra papilionaria*	Exp	1,00	6	Japan
Geometridae	*Gigantalcis flavolinearia*	Exp	0,25	7	Japan
Geometridae	*Hemithea aestivaria*	Exp	0,31	4	Czech Republic
Geometridae	*Hemithea aestivaria*	Exp	0,73	10	Japan
Geometridae	*Hydrelia sylvata*	Exp	0,31	15	Japan
Geometridae	*Hypagyrtis unipunctata*	Exp	0,05	67	USA
Geometridae	*Hypomecis lunifera*	Exp	0,34	4	Japan
Geometridae	*Hypomecis punctinalis*	Exp	0,55	14	Czech Republic
Geometridae	*Inurois fletcheri*	Exp	1,00	11	Japan
Geometridae	*Inurois fumosa*	Exp	0,00	278	Japan
Geometridae	*Inurois punctigera*	Exp	0,65	45	Japan
Geometridae	*Inurois tenuis*	Exp	0,44	249	Japan
Geometridae	*Iridopsis defectaria*	Exp	-0,42	7	USA
Geometridae	*Lambdina fervidaria*	Exp	0,20	36	USA
Geometridae	*Lomographa simplicior*	Exp	0,09	1137	Japan
Geometridae	*Lomographa vestaliata*	Exp	1,00	5	USA
Geometridae	*Lycia hirtaria*	Exp	-0,79	42	Czech Republic
Geometridae	*Macaria aemulataria*	Exp	1,00	6	USA
Geometridae	*Melanolophia signataria*	Exp	-0,58	58	USA
Geometridae	*Nemoria bistriaria*	Exp	0,94	3	USA
Geometridae	*Operophtera brumata*	Exp	0,63	3303	Czech Republic
Geometridae	*Operophtera brunnea*	Exp	0,03	2590	Japan
Geometridae	*Operophtera relegata*	Exp	0,24	114	Japan
Geometridae	*Operophtera bruceata*	Exp	0,23	5	USA
Geometridae	*Parectropis similaria*	Exp	0,24	11	Czech Republic
Geometridae	*Phaeoura quernaria*	Exp	0,44	3	USA

Geometridae	*Phigalia pilosaria*	Exp	0,14	52	Czech Republic
Geometridae	*Phigalia* sp. 1	Exp	0,21	11	Japan
Geometridae	*Phigalia verecundaria*	Exp	0,08	116	Japan
Geometridae	*Plagodis alcoolaria*	Exp	-0,27	3	USA
Geometridae	*Plagodis dolabraria*	Exp	0,55	4	Czech Republic
Geometridae	*Plemyria rubiginata*	Exp	-0,12	11	Japan
Geometridae	*Protoboarmia porcelaria*	Exp	0,28	8	USA
Geometridae	*Pseuderannis amplipennis*	Exp	-0,35	27	Japan
Geometridae	*Ramobia basifuscaria*	Exp	0,81	182	Japan
Geometridae	*Selenia tetralunaria*	Exp	0,25	14	Czech Republic
Geometridae	*Selenia tetralunaria*	Exp	0,61	12	Japan
Geometridae	*Trichopterigia* sp. 1	Exp	1,00	10	Japan
Geometridae	*Trichopteryx fastuosa*	Exp	0,49	66	Japan
Geometridae	*Trichopteryx ussurica*	Exp	1,00	3	Japan
Geometridae	*Trichopteryx ustata*	Exp	0,22	31	Japan
Gracillariidae	*Caloptilia betulicola*	She	0,81	12	Japan
Gracillariidae	*Caloptilia bimaculatella*	She	1,00	3	USA
Gracillariidae	*Caloptilia hemidactylella*	She	1	7	Czech Republic
Gracillariidae	*Caloptilia ostryaeella*	She	1,00	5	USA
Gracillariidae	*Caloptilia paradoxa*	She	0,98	8	USA
Gracillariidae	*Caloptilia semifascia*	She	1	5	Czech Republic
Hesperiidae	*Erynnis juvenalis*	She	0,65	23	USA
Lasiocampidae	*Malacosoma americanum*	She	-0,29	3	USA
Lasiocampidae	*Malacosoma disstria*	She	-0,20	8	USA
Lasiocampidae	*Malacosoma neustrium*	She	0,84	50	Czech Republic
Lasiocampidae	*Malacosoma neustrium*	She	0,38	7	Japan
Lecithoceridae	*Rhizosthenes falciformis*	She	0,41	141	Japan
Lecithoceridae	*Scythropiodes issikii*	She	1,00	4	Japan
Lecithoceridae	*Scythropiodes lividula*	She	0,11	13	Japan
Limacodidae	*Adoneta bicaudata*	Exp	1,00	5	USA
Limacodidae	*Apoda limacodes*	Exp	1	6	Czech Republic
Limacodidae	*Apoda y-inversum*	Exp	1,00	4	USA
Limacodidae	*Austrapoda hepatica*	Exp	0,29	3	Japan
Limacodidae	*Euclea delphinii*	Exp	0,32	8	USA
Limacodidae	*Isa textula*	Exp	1,00	6	USA
Limacodidae	*Lithacodes fasciola*	Exp	0,43	3	USA
Limacodidae	*Natada nasoni*	Exp	0,32	13	USA
Limacodidae	*Parasa chloris*	Exp	0,52	5	USA
Limacodidae	*Parasa sinica*	Exp	0,36	4	Japan
Lycaenidae	*Neozephyrus quercus*	Exp	0,96	20	Czech Republic
Lycaenidae	*Satyrium calanus*	Exp	0,46	4	USA
Megalopygidae	*Megalopyge crispata*	Exp	-0,13	3	USA
Mimallonidae	*Lacosoma chiridota*	She	0,95	6	USA
Noctuidae	*Achatia distincta*	Exp	0,10	14	USA
Noctuidae	*Acronicta afflicta*	Exp	0,45	7	USA
Noctuidae	*Acronicta americana*	Exp	1,00	11	USA
Noctuidae	*Acronicta haesitata*	Exp	0,99	22	USA
Noctuidae	*Acronicta hasta*	Exp	1,00	3	USA
Noctuidae	*Acronicta impleta*	Exp	-0,12	20	USA
Noctuidae	*Acronicta interrupta*	Exp	1,00	3	USA

Noctuidae	*Acronicta lobeliae*	Exp	1,00	4	USA
Noctuidae	*Acronicta modica*	Exp	0,96	8	USA
Noctuidae	*Acronicta ovata*	Exp	0,79	18	USA
Noctuidae	*Acronicta retardata*	Exp	1,00	3	USA
Noctuidae	*Acronicta tristis*	Exp	1,00	26	USA
Noctuidae	*Amphipyra berbera*	Exp	0,02	11	Czech Republic
Noctuidae	*Amphipyra pyramidea*	Exp	0,78	13	Czech Republic
Noctuidae	*Amphipyra pyramidea*	Exp	0,09	26	Japan
Noctuidae	*Amphipyra pyramidoides*	Exp	0,04	13	USA
Noctuidae	*Anorthoa munda*	Exp	-0,15	40	Japan
Noctuidae	*Asteroscopus sphinx*	Exp	0,43	18	Czech Republic
Noctuidae	*Belciades niveola*	Exp	0,58	5	Japan
Noctuidae	*Brachionycha nubeculosa*	Exp	0,52	6	Japan
Noctuidae	*Charadra deridens*	Exp	0,94	3	USA
Noctuidae	*Chasminodes atratus*	She	0,72	33	Japan
Noctuidae	*Chasminodes cilia*	She	1,00	14	Japan
Noctuidae	*Chasminodes* sp. 1	She	0,41	86	Japan
Noctuidae	*Chasminodes sugii*	She	1,00	3	Japan
Noctuidae	*Colocasia coryli*	Exp	0,16	5	Czech Republic
Noctuidae	*Comachara cadburyi*	Exp	1,00	201	USA
Noctuidae	*Conistra grisescens*	Exp	0,13	72	Japan
Noctuidae	*Conistra rubiginosa*	Exp	0,46	35	Czech Republic
Noctuidae	*Conistra* sp. 3	Exp	-0,38	104	Japan
Noctuidae	*Conistra* sp. 4	Exp	0,68	8	Japan
Noctuidae	*Conistra vaccinii*	Exp	0,61	6	Czech Republic
Noctuidae	*Cosmia affinis*	Exp	1	4	Czech Republic
Noctuidae	*Cosmia affinis*	Exp	0,59	7	Japan
Noctuidae	*Cosmia moderata*	Exp	-0,16	6	Japan
Noctuidae	*Cosmia trapezina*	Exp	0,47	110	Czech Republic
Noctuidae	*Cosmia trapezina*	Exp	0,15	166	Japan
Noctuidae	*Cosmia unicolor*	Exp	0,11	48	Japan
Noctuidae	*Craniophora ligustri*	Exp	0,99	25	Czech Republic
Noctuidae	*Craniophora* sp. 1	Exp	0,90	3	Japan
Noctuidae	*Crocigrapha normani*	Exp	0,06	3	USA
Noctuidae	*Daseochaeta viridis*	Exp	0,07	5	Japan
Noctuidae	*Diadochia* sp. 2	Exp	0,90	30	Japan
Noctuidae	*Dryobotodes eremita*	Exp	0,88	166	Czech Republic
Noctuidae	*Egira* sp. 1	Exp	-0,24	46	Japan
Noctuidae	*Eremohadena* sp. 14	Exp	0,27	7	Japan
Noctuidae	*Eupsilia morrisoni*	Exp	0,64	8	USA
Noctuidae	*Eupsilia transversa*	Exp	0,87	41	Czech Republic
Noctuidae	*Himella fidelis*	Exp	0,34	44	USA
Noctuidae	*Lithophane antennata*	Exp	-0,15	25	USA
Noctuidae	*Lithophane ornitopus*	Exp	0,88	20	Czech Republic
Noctuidae	*Lithophane plumbearis*	Exp	0,62	20	Japan
Noctuidae	*Lithophane semibrunnea*	Exp	1	20	Czech Republic
Noctuidae	*Lithophane* sp. 1	Exp	0,71	9	Japan
Noctuidae	*Morrisonia confusa*	She	0,01	59	USA
Noctuidae	*Morrisonia latex*	She	0,38	11	USA
Noctuidae	*Orthosia carnipennis*	Exp	0,20	107	Japan

Noctuidae	*Orthosia cerasi*	Exp	0,16	153	Czech Republic
Noctuidae	*Orthosia cruda*	Exp	0,46	544	Czech Republic
Noctuidae	*Orthosia gracilis*	Exp	1	106	Czech Republic
Noctuidae	*Orthosia hibisci*	Exp	-0,40	8	USA
Noctuidae	*Orthosia incerta*	Exp	-0,15	12	Czech Republic
Noctuidae	*Orthosia odiosa*	Exp	0,36	102	Japan
Noctuidae	*Orthosia* sp. 1	Exp	-0,20	16	Japan
Noctuidae	*Perigrapha munda*	Exp	-0,32	25	Czech Republic
Noctuidae	*Polygrammate hebraeicum*	Exp	1,00	6	USA
Noctuidae	*Psaphida resumens*	Exp	0,47	3	USA
Noctuidae	*Psaphida rolandi*	Exp	1,00	17	USA
Nolidae	*Baileya levitans*	Exp	1,00	4	USA
Nolidae	*Baileya ophthalmica*	Exp	0,67	12	USA
Nolidae	*Bena bicolorana*	Exp	0,91	28	Czech Republic
Nolidae	*Meganola phylla*	Exp	0,94	3	USA
Nolidae	*Meganola strigulosa*	Exp	0,35	4	Japan
Nolidae	*Pseudoips prasinana*	Exp	0,92	28	Czech Republic
Nolidae	*Sinna extrema*	Exp	0,25	60	Japan
Notodontidae	*Cnethodonta gisescens*	Exp	0,97	3	Japan
Notodontidae	*Heterocampa guttivita*	Exp	-0,14	29	USA
Notodontidae	*Himeropteryx miraculosa*	Exp	0,60	58	Japan
Notodontidae	*Hyperaeschra georgica*	Exp	0,96	7	USA
Notodontidae	*Lochmaeus bilineata*	Exp	0,41	5	USA
Notodontidae	*Lophocosma sarantuja*	Exp	0,97	4	Japan
Notodontidae	*Macrurocampa marthesia*	Exp	0,97	40	USA
Notodontidae	*Nadata gibbosa*	Exp	0,96	45	USA
Notodontidae	*Nerice bidentata*	Exp	1,00	3	USA
Notodontidae	*Nerice bipartita*	Exp	1,00	3	Japan
Notodontidae	*Ptilodon capucina*	Exp	0,44	4	Czech Republic
Notodontidae	*Ptilodon cucullina*	Exp	1	3	Czech Republic
Notodontidae	*Ptilodon* sp. 1	Exp	1,00	4	Japan
Notodontidae	*Ptilophora plumigera*	Exp	1	186	Czech Republic
Notodontidae	*Ptilophora* sp. 2	Exp	1,00	15	Japan
Notodontidae	*Schizura leptinoides*	Exp	0,57	3	USA
Notodontidae	*Semidonta biloba*	Exp	0,96	14	Japan
Notodontidae	*Symmerista albifrons*	Exp	0,96	167	USA
Oecophoridae	*Carcina quercana*	She	0,33	33	Czech Republic
Psychidae	*Bacotia claustrella*	She	-0,07	27	Czech Republic
Psychidae	*Dahlica lichenella*	She	1	14	Czech Republic
Psychidae	*Dahlica triquetrella*	She	0,76	19	Czech Republic
Psychidae	*Diplodoma laichartingella*	She	1	4	Czech Republic
Psychidae	*Proutia betulina*	She	0,11	19	Czech Republic
Psychidae	*Psyche casta*	She	-0,62	10	Czech Republic
Psychidae	*Sterrhopterix fusca*	She	0,00	8	Czech Republic
Psychidae	*Taleporia tubulosa*	She	0,82	16	Czech Republic
Pyralidae	*Acrobasis repandana*	She	0,98	16	Czech Republic
Pyralidae	*Actrix* sp. ACF5305	She	1,00	5	USA
Pyralidae	*Oneida lunulalis*	She	0,03	7	USA
Pyralidae	*Phycita roborella*	She	0,74	31	Czech Republic
Pyralidae	*Pococera* sp. AAA3814	She	0,95	5	USA

Pyralidae	*Pococera* sp. AAA4979	She	0,96	6	USA
Pyralidae	*Pococera* sp. ABY6852	She	0,96	10	USA
Pyralidae	*Pococera* sp. ACE4466	She	0,60	78	USA
Pyralidae	*Pococera* sp. ACF4182	She	0,99	11	USA
Pyralidae	*Salebriaria engeli*	She	1,00	7	USA
Pyralidae	*Salebriaria tenebrosella*	She	1,00	5	USA
Pyralidae	*Termioptycha bilineata*	She	0,46	5	Japan
Roeslerstammiidae	*Roeslerstammia erxlebella*	She	1	12	Czech Republic
Saturniidae	*Actias luna*	Exp	0,01	4	USA
Saturniidae	*Aglia japonica*	Exp	1,00	23	Japan
Saturniidae	*Anisota senatoria*	Exp	1,00	132	USA
Saturniidae	*Dryocampa rubicunda*	Exp	1,00	13	USA
Saturniidae	*Saturnia jonasii*	Exp	-0,54	33	Japan
Sphingidae	*Amorpha juglandis*	Exp	0,98	8	USA
Sphingidae	*Ceratomia undulosa*	Exp	1,00	5	USA
Sphingidae	*Marumba jankowskii*	Exp	0,68	29	Japan
Sphingidae	*Meganoton analis*	Exp	0,49	17	Japan
Tortricidae	*Acleris aurichalcana*	She	1,00	4	Japan
Tortricidae	*Acleris chalybeana*	She	1,00	4	USA
Tortricidae	*Acleris cristana*	She	0,65	11	Japan
Tortricidae	*Acleris delicatana*	She	0,94	242	Japan
Tortricidae	*Acleris dentata*	She	0,76	11	Japan
Tortricidae	*Acleris elegans*	She	0,95	57	Japan
Tortricidae	*Acleris filipjevi*	She	0,57	6	Japan
Tortricidae	*Acleris flavivittana*	She	1,00	5	USA
Tortricidae	*Acleris forsskaleana*	She	0,83	32	Czech Republic
Tortricidae	*Acleris nivisellana*	She	1,00	3	USA
Tortricidae	*Acleris paradiseana*	She	0,49	37	Japan
Tortricidae	*Acleris pulchella*	She	0,12	3	Japan
Tortricidae	*Acleris* sp. 1	She	1,00	8	Japan
Tortricidae	*Acleris* sp. 2	She	1,00	7	Japan
Tortricidae	*Acleris sparsana*	She	1	4	Czech Republic
Tortricidae	*Ancylis* sp. AAA8534	She	0,96	65	USA
Tortricidae	*Archips crataegana*	She	-0,35	174	Japan
Tortricidae	*Archips fuscocupreanus*	She	-0,35	32	Japan
Tortricidae	*Archips nigricaudana*	She	0,05	251	Japan
Tortricidae	*Archips podana*	She	0,08	7	Czech Republic
Tortricidae	*Archips xylosteana*	She	0,20	7	Czech Republic
Tortricidae	*Archips xylosteana*	She	-0,16	16	Japan
Tortricidae	*Argyrotaenia alisellana*	She	0,96	10	USA
Tortricidae	*Argyrotaenia mariana*	She	0,11	5	USA
Tortricidae	*Argyrotaenia quercifoliana*	She	0,83	53	USA
Tortricidae	*Argyrotaenia velutinana*	She	-0,31	6	USA
Tortricidae	*Cenopis directana*	She	-0,06	11	USA
Tortricidae	*Choristoneura adumbratana*	She	-0,45	22	Japan
Tortricidae	*Choristoneura diversana*	She	0,16	25	Czech Republic
Tortricidae	*Choristoneura hebenstreitella*	She	0,28	4	Czech Republic
Tortricidae	*Choristoneura longicellana*	She	0,19	6	Japan
Tortricidae	*Choristoneura rosaceana*	She	-0,18	25	USA
Tortricidae	*Epinotia exquisitana*	She	0,20	79	Japan

Tortricidae	*Epinotia* sp. 1	She	1,00	68	Japan
Tortricidae	*Eudemis brevisetosa*	She	0,45	8	Japan
Tortricidae	*Eudemis porphyrana*	She	1,00	214	Czech Republic
Tortricidae	*Eudemis porphyrana*	She	0,77	249	Japan
Tortricidae	*Eudemis profundana*	She	1	10	Czech Republic
Tortricidae	*Gretchena deludana*	She	0,99	161	USA
Tortricidae	*Gypsonoma* sp. 1	She	1,00	10	Japan
Tortricidae	*Hedya semiassana*	She	1,00	3	Japan
Tortricidae	*Hendecaneura impar*	She	0,84	34	Japan
Tortricidae	*Homonopsis foederatana*	She	1,00	11	Japan
Tortricidae	*Homonopsis illotana*	She	-0,72	32	Japan
Tortricidae	*Olethreutes mori*	She	0,97	387	Japan
Tortricidae	*Olethreutes subretracta*	She	0,06	14	Japan
Tortricidae	*Pammene giganteana*	She	0,59	12	Czech Republic
Tortricidae	*Pandemis cerasana*	She	-0,03	7	Czech Republic
Tortricidae	*Pandemis corylana*	She	0,31	15	Czech Republic
Tortricidae	*Pandemis corylana*	She	-0,09	3	Japan
Tortricidae	*Pandemis heparana*	She	0,14	7	Czech Republic
Tortricidae	*Pandemis limitata*	She	0,96	4	USA
Tortricidae	*Phaecasiophora niveiguttana*	She	1,00	7	USA
Tortricidae	*Pseudexentera cressoniana*	She	0,98	20	USA
Tortricidae	*Pseudexentera haracana*	She	0,98	143	USA
Tortricidae	*Pseudexentera hodsoni*	She	0,77	7	USA
Tortricidae	*Pseudexentera oregonana*	She	1,00	17	USA
Tortricidae	*Pseudexentera sepia*	She	1,00	6	USA
Tortricidae	*Pseudexentera spoliana*	She	0,98	35	USA
Tortricidae	*Pseudohedya cincinna*	She	0,73	50	Japan
Tortricidae	*Pseudohedya dentata*	She	0,81	326	Japan
Tortricidae	*Pseudohedya gradana*	She	0,76	353	Japan
Tortricidae	*Pseudohedya retracta*	She	0,89	359	Japan
Tortricidae	*Pseudohedya satoi*	She	0,92	160	Japan
Tortricidae	*Pseudohedya* sp. 1	She	1,00	3	Japan
Tortricidae	*Ptycholoma imitator*	She	-0,06	4	Japan
Tortricidae	*Ptycholoma lecheana*	She	0,03	17	Japan
Tortricidae	*Rhopobota naevana*	She	0,85	733	Japan
Tortricidae	*Rhopobota* sp. 2	She	1,00	148	Japan
Tortricidae	*Rhopobota* sp. 6	She	1,00	28	Japan
Tortricidae	*Sereda tautana*	She	0,41	7	USA
Tortricidae	*Spilonota ocellana*	She	0,95	3	Czech Republic
Tortricidae	*Tortricoides alternella*	She	0,98	74	Czech Republic
Tortricidae	*Tortrix viridana*	She	0,88	28	Czech Republic
Tortricidae	*Zeiraphera isertana*	She	1	112	Czech Republic
Tortricidae	*Zeiraphera shimekii*	She	-0,11	12	Japan
Tortricidae	*Zeiraphera varinea*	She	0,94	28	Japan
Yponomeutidae	*Argyresthia alpha*	She	0,03	10	Japan
Yponomeutidae	*Yponomeuta refrigerata*	She	1,00	311	Japan
Ypsolophidae	*Ypsolopha alpella*	Exp	0,95	5	Czech Republic
Ypsolophidae	*Ypsolopha lucella*	Exp	1	6	Czech Republic
Ypsolophidae	*Ypsolopha parenthesella*	Exp	1	4	Czech Republic
Ypsolophidae	*Ypsolopha parenthesella*	She	0,58	24	Japan

Ypsolophidae	*Ypsolopha sequella*	Exp	1	10	Czech Republic
Ypsolophidae	*Ypsolopha ustella*	Exp	0,82	23	Czech Republic

Table S4 Comparison of different indices (incidence and abundance-based) on the relationship between host plant phylogenetic distance and similarity of the caterpillar assemblages. Pearson's product-moment correlation coefficients and their significance values are represented across guilds as well as separately for exposed feeders and shelter builders (modified Mantel tests; 9999 permutations).

**Index**	**Across guilds**		**Exposed feeders**		**Shelter builders**	
	r	*P*	*r*	*P*	*r*	*P*
Jaccard	-0.23	< 0.001	-0.13	< 0.001	-0.34	< 0.001
Bray-Curtis	-0.23	< 0.001	-0.13	0.003	-0.22	0.043
Morisita-Horn	-0.36	< 0.001	-0.23	< 0.001	-0.28	0.004

Table S5 Linear mixed models (LMMs) investigating the influence of plant phylogenetic isolation (PI), feeding guild (Guild), resource availability (Leaf area) and locality (Site) on abundance, density, richness, and mean specialisation of caterpillar assemblages. Model performance is shown based on their AICc values, ΔAICc units, and their weights (*w*). The best models with ΔAICc ≤ 2 are presented in bold.

**Model**		**Abundance**			**Density**			**Richness**			**Specialisation (DSI*)**		
	d.f.	AICc	ΔAICc	*w*	AICc	ΔAICc	*w*	AICc	ΔAICc	*w*	AICc	ΔAICc	*w*
~ (1|Tree ID)	3	330.2.	75.2	<0.001	184.2	37.3	<0.001	682.5	123.8	<0.001	3.1	31.2	<0.001
~ Site + (1|Tree ID)	5	329.1	74.2	<0.001	161.4	14.5	<0.001	683.9	125.2	<0.001	6.7	34.9	<0.001
~ Guild + (1|Tree ID)	4	324.9	69.9	<0.001	179.5	32.6	<0.001	631.2	72.4	<0.001	-14.6	13.6	<0.001
~ PI + (1|Tree ID)	4	324.0	69.0	<0.001	184.8	37.9	<0.001	674.2	115.4	<0.001	-3.8	24.4	<0.001
~ Leaf area + (1|Tree ID)	4	302.3	47.3	<0.001	185.3	38.4	<0.001	634.9	76.2	<0.001	-0.7	27.4	<0.001
~ Site + Guild + (1|Tree ID)	6	324.0	69.0	<0.001	156.9	10.0	0.003	632.7	73.9	<0.001	-10.8	17.3	<0.001
~ PI + Guild + (1|Tree ID)	5	318.8	63.8	<0.001	180.2	33.3	<0.001	622.9	64.1	<0.001	-21.4	6.8	0.018
~ PI + Site + (1|Tree ID)	6	321.0	66.0	<0.001	161.9	15.0	<0.001	673.0	114.2	<0.001	-1.0	27.1	<0.001
~ Leaf area + Site + (1|Tree ID)	6	281.4	26.4	<0.001	163.7	16.8	<0.001	620.7	61.9	<0.001	3.6	31.7	<0.001
~ Leaf area + Guild + (1|Tree ID)	5	297.1	42.1	<0.001	180.7	33.8	<0.001	583.6	24.9	<0.001	-18.3	9.8	0.004
~ PI + Leaf area + (1|Tree ID)	5	301.4	46.5	<0.001	184,5	37.6	<0.001	631.9	73.1	<0.001	-4.2	23.9	<0.001
~ Site*Guild + (1|Tree ID)	8	302.6	47.6	<0.001	**146.9**	**0**	**0.438**	625.9	67.1	<0.001	-20.6	7.6	0.012
~ PI + Site + Guild + (1|Tree ID)	7	315.9	60.9	<0.001	157.4	10.5	0.002	621.8	63.0	<0.001	-18.5	9.7	0.004
~ PI + Site*Guild + (1|Tree ID)	9	294.6	39.6	<0.001	**147.5**	**0.6**	**0.321**	615.1	56.3	<0.001	**-28.2**	**0**	**0.546**
~ Leaf area + Site + Guild + (1|Tree ID)	7	276.3	21.3	<0.001	159.2	12.3	<0.001	567.9	9.1	0.008	-13.9	14.2	<0.001
~ Leaf area + Site*Guild + (1|Tree ID)	9	**255.0**	**0**	**0.550**	149.4	2.5	0.126	561.2	2.4	0.222	-23.6	4.6	0.056
~ PI + Leaf area + Guild + (1|Tree ID)	6	296.3	41.3	<0.001	180.0	33.1	<0.001	580.6	21.8	<0.001	-21.8	6.4	0.023
~ PI + Leaf area + Guild + Site + (1|Tree ID)	8	276.5	21.5	<0.001	159.5	12.6	<0.001	565.4	6.6	0.028	-17.6	10.5	0.002
~ PI + Leaf area + Guild*Site + (1|Tree ID)	10	**255.4**	**0.4**	**0.450**	149.8	2.9	0.105	**558.8**	**0**	**0.742**	**-27.2**	**1.0**	**0.331**

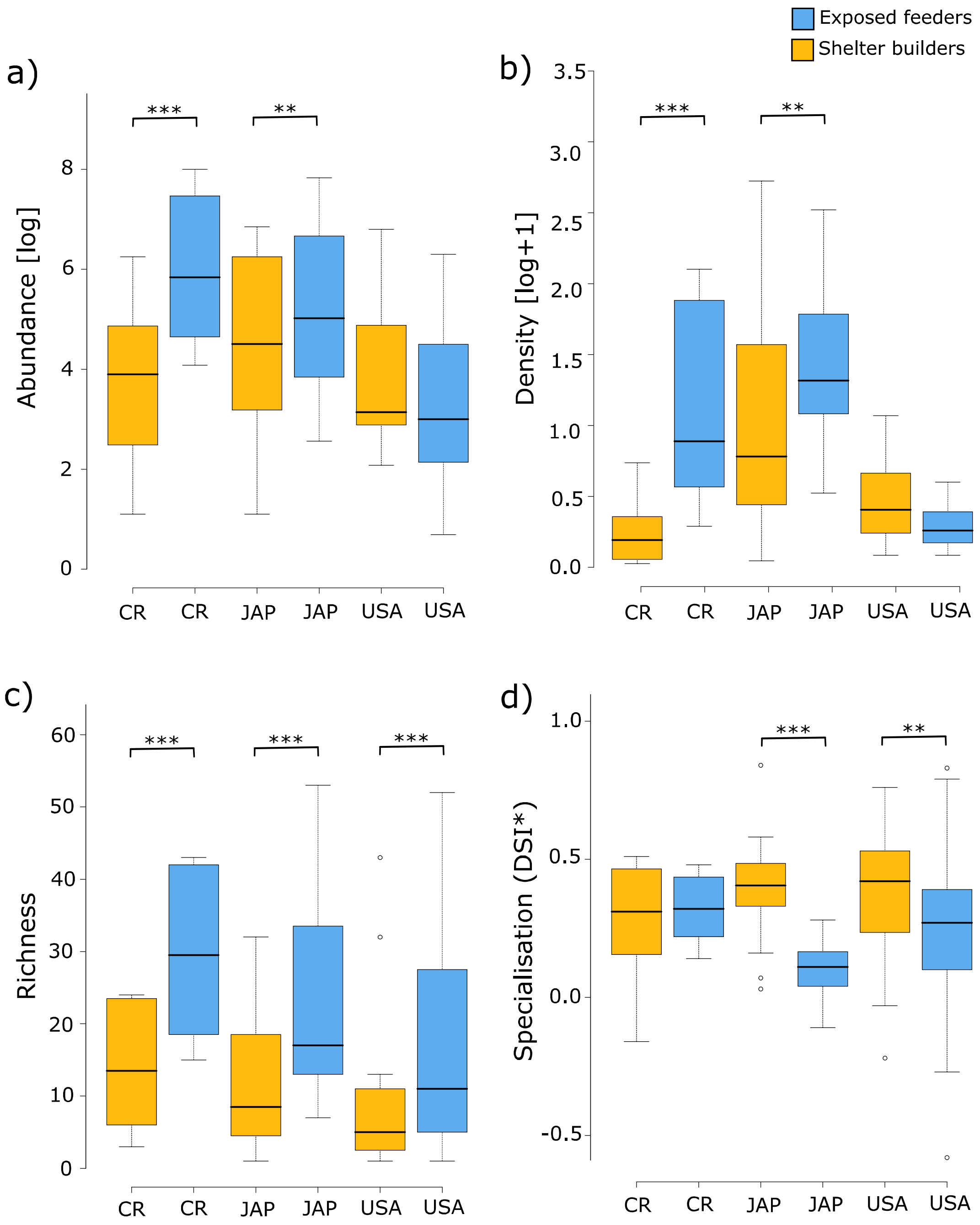

Figure S1 Comparisons of exposed feeding and shelter-building caterpillar assemblages regarding their **a)** abundance, **b)** density, **c)** richness, and **d)** mean specialisation per host plant species in the three sampling sites (CZ = Lanžhot, Czech Republic; Jap = Tomakomai, Japan; USA = Toms Brook, United States of America). Asterisks indicate significance values derived from Tukey post-hoc tests on the best fitting LMMs: *$P < 0.05$, **$P < 0.01$, ***$P < 0.001$.
